# The RGG domain in the C-terminus of the DEAD box helicases Dbp2 and Ded1 is necessary for G-quadruplex destabilization

**DOI:** 10.1093/nar/gkab620

**Published:** 2021-07-24

**Authors:** Kevin Kok-Phen Yan, Ikenna Obi, Nasim Sabouri

**Affiliations:** Department of Medical Biochemistry and Biophysics, Umeå University, 901 87 Umeå, Sweden; Department of Medical Biochemistry and Biophysics, Umeå University, 901 87 Umeå, Sweden; Department of Medical Biochemistry and Biophysics, Umeå University, 901 87 Umeå, Sweden

## Abstract

The identification of G-quadruplex (G4) binding proteins and insights into their mechanism of action are important for understanding the regulatory functions of G4 structures. Here, we performed an unbiased affinity-purification assay coupled with mass spectrometry and identified 30 putative G4 binding proteins from the fission yeast *Schizosaccharomyces pombe*. Gene ontology analysis of the molecular functions enriched in this pull-down assay included mRNA binding, RNA helicase activity, and translation regulator activity. We focused this study on three of the identified proteins that possessed putative arginine-glycine-glycine (RGG) domains, namely the Stm1 homolog Oga1 and the DEAD box RNA helicases Dbp2 and Ded1. We found that Oga1, Dbp2, and Ded1 bound to both DNA and RNA G4s *in vitro*. Both Dbp2 and Ded1 bound to G4 structures through the RGG domain located in the C-terminal region of the helicases, and point mutations in this domain weakened the G4 binding properties of the helicases. Dbp2 and Ded1 destabilized less thermostable G4 RNA and DNA structures, and this ability was independent of ATP but dependent on the RGG domain. Our study provides the first evidence that the RGG motifs in DEAD box helicases are necessary for both G4 binding and G4 destabilization.

## INTRODUCTION

G-quadruplexes (G4s) are non-canonical higher-ordered nucleic acid secondary structures characterized by square planar configurations of four guanines held together through Hoogsteen-type hydrogen bonding. The G4 structure is stabilized by a central cation, and based on the nature of this ion, the loop length and base composition, the strand orientation, and the molecularity, the G4 folding process is highly dynamic and heterogeneous (1,2). Indeed, G4 structures come in many different structural variants, such as two or more stacks of G-tetrads, intra- and intermolecular structures, and different parallel and antiparallel topologies (3). Their formation *in vivo* as well as their significance was often questioned in the past, but increasing evidence of G4 folding in nucleic acids in cells support their existence (4,5). For instance, G4-specific antibodies recognize G4 structures in human cells (6–9). Also, cell permeable G4-selective small molecules have been developed that light up G4 structures in living cells (10–15). The evolutionary conservation and enrichment of G4 structures in a wide variety of organisms at specific genomic locations (16–18), their formation in both DNA and RNA (6,9,12,19–21), and their presence in both nuclear and mitochondrial genomes (14,22) strongly suggest that G4 structures play important biological roles (23). These roles include the regulation of replication, transcription, and translation as well as the maintenance of telomeres (24–26).

To understand the mechanisms through which G4s play their roles, affinity purification methods with human and *Saccharomyces cerevisiae* cell lysates using G4 DNA structures as baits have attempted to identify and characterize novel G4-binding proteins (27–30). The DEAH-box helicase 36 (DHX36) gene product was one of the first helicases to be isolated and characterized using these affinity purification methods (27). Additional helicases that are specialized G4 unwinders include the breast cancer-associated Pif1 family helicases and the Fanconi anemia group protein J (FANCJ) helicase, and these promote genome integrity by resolving G4 structures that form during DNA replication (31–37). Furthermore, G4-binding proteins like Ewing's sarcoma (EWS), nucleolar RNA helicase 2 (DDX21), and SRA stem-loop-interacting RNA-binding protein (SLIRP) bind to G4 structures and play a role in controlling protein expression through either transcription or translation-related processes (38–41).

The specific domains that recognize G4 structures have not been determined for most G4-binding proteins; however, the crystal structure of a bacterial RecQ helicase showed that a guanine-specific binding pocket is important for G4 destabilization (42). Other types of domains that bind G4s are the RNA recognition motif (RRM) of SLIRP and the HEAT-like α-helical repeats (HEAT repeats) in replication timing regulatory factor 1 (Rif1) (40,43). The arginine-glycine-glycine (RGG) motif has also emerged as one of the protein domains involved in G4 binding (44–46). Proteins harboring two or three repeats of RGG or RG have been reported to affect various cellular mechanisms, such as splicing, transcription, DNA damage signaling, and translation (44). However, this motif is far from being a generalized G4-binding motif. A typical RGG/RG domain is composed of two or three repeats of RGG or RG, with up to four residues between each repeat (44). It has been shown that the positively charged arginine in the repeats and the aromatic residues between the repeats are important key features for G4 binding (46), and it is suggested that these residues enable either electrostatic or π-stacking interactions with the G-tetrad and thereby enhance the protein's binding affinity for G4s (46).

The fission yeast *Schizosaccharomyces pombe* is one of the model organisms used to study G4s and their impact on genome stability. About 450 predicted three G-tetrad stacked G4 structures have been identified in the non-repetitive regions of the *S. pombe* genome, with significant enrichment in promoters, untranslated regions (UTRs), nucleosome-depleted regions, rDNA, and telomeres (36). The *S. pombe* Pfh1 and Rif1 proteins bind to G4 structures *in vitro* (47–49). Pfh1 is a 5′–3′ helicase from the Pif1 family that resolves G4 DNA structures and thus ensures genomic stability (36,47,50–51), while Rif1 is a telomere-associated protein that also has a role in regulating replication timing (49,52–53). In this study, we sought to identify new G4-binding proteins from *S. pombe* and thus to contribute to enhancing our knowledge about the G4 interactome and to provide insights into the relevance of G4s in different species. By using an unbiased pull-down assay combined with a proteomics approach, we identified 30 novel G4-binding proteins. We focused on three of these hits that possessed putative RGG domains—Oga1, Ded1 and Dbp2—and performed in-depth biochemical characterization of these proteins. By performing mutational studies, we showed the importance of the RGG domain in G4 recognition and destabilization by the DEAD box helicases, Ded1 and Dbp2, and shed new light on the evolutionary conservation of this domain in G4 biology.

## MATERIALS AND METHODS

### Preparation of DNA and RNA G4s

All oligonucleotides used in this study are described in Table 1, Table 2 and Supplementary Table S1. High purity salt-free oligonucleotides were purchased from Eurofins. G4-forming oligonucleotides at a concentration of 50 μM were folded by heating for 5 min at 95°C in 10 mM Tris–HCl pH 7.5 and either 100 mM NaCl or 100 mM KCl followed by cooling overnight to room temperature.

**Table 1.** Design of the oligonucleotides used for affinity purification of G4-binding proteins. Wild type and mutated G-tracts are underlined and in bold. The mutated control oligonucleotides (m4) have one G to T substitution in each G-tract indicated as ‘t’. A scrambled (scr) G-rich sequence was used as an additional control

Name	5′end modification	Sequence
rDNA G4	Biotin-TEG	5′-**GGGG**AA**GGG**T**GGGG**CATGTTAT**GGG**-3′
rDNA scr	Biotin-TEG	5′-GGTGCGAGGTGAGTGTGGAGTGAGG-3′
rDNA m4	Biotin-TEG	5′-**GtGG**AA**GtG**T**GGtG**CATGTTAT**GtG**-3′
10A-rDNA G4	Biotin-TEG	5′-AAAAAAAAAA**GGGG**AA**GGG**T**GGGG**CATGTTAT**GGG**-3′
10A-rDNA scr	Biotin-TEG	5′-AAAAAAAAAAGGTGCGAGGTGAGTGTGGAGTGAGG-3′
10A-rDNA m4	Biotin-TEG	5′-AAAAAAAAAA**GtGG**AA**GtG**T**GGtG**CATGTTAT**GtG**-3′

**Table 2.** Sequences of the oligonucleotides used for the EMSA and helicase assays. The ^32^P end-labeling on the 5′ end is represented by a star. G-tracts are underlined

Name	Type	Sequence
10A-rDNA G4	DNA	*5′-AAAAAAAAAAGGGGAAGGGTGGGGCATGTTATGGG-3′
10A-rDNA scr	DNA	*5′-AAAAAAAAAAGGTGCGAGGTGAGTGTGGAGTGAGG-3′
10A-rDNA m4	DNA	*5′-AAAAAAAAAAGtGGAAGtGTGGtGCATGTTATGtG-3′
c-myc G4	DNA	*5′-GGCCGCTTATGGGGAGGGTGGGGAGGGTGGGGAAGGTGGGGAGGAGACTCA-3′
c-myc scr	DNA	*5′-GGCGGCGGCGTGTGAGTGAGTGAGTGAGAGGTGAGGAGAGCGTGGCGGAGG-3′
Z33 G4	DNA	*5′-AAAGTGATGGTGGTGGGGGAAGGATTTTCGAAC-3′
5′-RNA-G4	RNA	*5′-AAAAAAAAAAGGGGAAGGGUGGGGCAUGUUAUGGG-3′
5′-RNA-G4-3′	RNA	*5′-UUUUUGGGGAAGGGUGGGGCAUGUUAUGGGUUUUU-3′
ssRNA	RNA	*5′-UUUUGUUUUGUUUUGUUUUGUUUUAAGCACCGUAAAGA-3′
dsRNA	RNA	5′-UCUUUACGGUGCUUAAAACAAAACAAAACAAAACAAAA-3′
		3′-AGAAAUGCCACGAAUUUUGUUUUGUUUUGUUUUGUUUU-5′*
5′-RNA	RNA	3′-UCGUGGCAUUUCU-5′*
		5′-AAAACAAAACAAAACAAAACAAAAUAGCACCGUAAAGA-3′
3′-RNA	RNA	3′-AGAAAUGCCACGA-5′*
		5′-UCUUUACGGUGCUUAAAACAAAACAAAACAAAACAAAA-3′
RNA-2G-G4	RNA	*5′-GUUGGUGGUGGUGGUGU-3′
DNA-2G-G4	DNA	*5′-GTTGGTGGTGGTGGTGT-3′
Trap	DNA	5′-ACACCACCACCACCAAC-3′
Anti-trap	DNA	5′-GTTGGTGGTGGTGGTGT-3′
DNA-3G-G4	DNA	*5′-TTTTTTTTTTTTTTTGAGGGTGGGTAGGGTGGGTAA-3′
Trap for DNA-3G-G4	DNA	5′-TTACCCACCCTACCCACCCTCA-3′
Anti-trap for DNA- 3G-G4	DNA	5′-TGAGGGTGGGTAGGGTGGGTAA-3′
Trap for 5′-RNA-G4	DNA	5′-CCCATAACATGCCCCACCCTTCCCC-3′
Anti-trap for 5′-RNA-G4	DNA	GGGGAAGGGTGGGGCATGTTATGGG

### Growth media

The Pombe Minimal Glutamate (PMG) media contained Dextrose (20 g/l), Phthalic Acid K+ (3 g/l), Na_2_HPO_4_ (2.2 g/l), L-glutamic acid (3.75 g/l), MgCl_2_.6H_2_O (1.05 g/l), CaCl_2_.2H_2_O (14.7 mg/l), KCl (1 g/l), Na_2_SO_4_ (40 mg/l), pantothenic acid (1 mg/l), nicotinic acid (10 mg/l), inositol (10 mg/l), Biotin (1 mg/l), H_3_BO_3_ (0.5 mg/l), MnSO_4_.H_2_O (0.4 mg/l), ZnSO_4_.7H_2_O (0.4 mg/l), FeCl_3_.6H_2_O (0.2 mg/l), Na_2_MoO_4_.2H_2_O (0.04 mg/l), KI (0.1 mg/l), CuSO_4_.5H_2_O (0.04 mg/l), citric acid (1 mg/l), adenine (50 mg/l), l-histidine HCl (50 mg/l), l-leucine (50 mg/l), l-lysine HCl (50 mg/l) and uracil (50 mg/l). EMM2 media has the same composition as the PMG media except that L-glutamic acid is replaced with NH_4_Cl (5 g/l).

### Circular dichroism

Samples containing 5 μM oligonucleotide in 10 mM Tris–HCl pH 7.5 and 100 mM NaCl or KCl were analyzed in a 1 mm path length quartz cuvette (Hellma) on a J-720 spectropolarimeter (JASCO). Measurements were recorded at 225–325 nm at either only 25°C, or at 25, 45, 65 and 85°C with the following parameters: 0.5 nm data pitch, continuous, 50 nm/min scanning speed, 1 s response, 2 nm band width, four spectra accumulations and 100 mdeg standard sensitivity. The background signal from the buffer was corrected using a blank containing no oligonucleotide.

### Preparation of yeast protein extracts

Yeast cells were grown to a density of 10^7^ cells/ml before harvesting and freezing at −80°C. The cell pellet was then ground in a freezer mill cryogenic grinder. The resulting protein powder was resuspended in SP lysis buffer (20 mM HEPES pH 7.9, 100 mM KAc, 2 mM MgCl_2_, 1 mM DTT, 3 mM EDTA, 0.1% igepal, 10% glycerol and cOmplete protease inhibitor (Roche)) and centrifuged at 20 000 × *g* for 15 min (JA-25.50, Beckman Coulter). The supernatant containing the total protein extract was then either stored at −80°C until use or further processed for enrichment of nuclear proteins. For nuclear protein enrichment, the sample was centrifuged for 90 min at 42 000 rpm (Ti45 rotor, Beckman Coulter). The lipid-rich layer and the soluble fraction were discarded, while the lower murky layer (the chromatin fraction) was washed with SP lysis buffer by centrifugation for 90 min at 38 000 rpm in an SW60 rotor (Beckman Coulter). The pellet was resuspended in SP lysis buffer (2 ml per 10 g of starting material) using a dounce homogenizer before adding NH_4_SO_4_ (500 mM final concentration). The solution was stirred for 30 min at 4°C, polyethylenemine was added to 0.1% final concentration, the solution was stirred for another 15 min at 4°C, and the solution was centrifuged for 90 min at 38 000 rpm (SW60 rotor). The resulting supernatant (nuclear protein extract) was then stored at −80°C until use.

### Pull-down experiment

A total of 3 nmol of folded biotinylated oligonucleotides was incubated for 1 h at 4°C with 0.5 mg of total or nuclear protein extract in a volume of 500 μl of SP lysis buffer. A total of 200 μl of neutravidin beads (Pierce High Capacity NeutrAvidin Agarose, Thermo Fisher) were washed in SP lysis buffer then added to the sample and incubated for 1 h at 4°C to pull down the bound proteins. The beads were recovered by centrifugation (1 min at 2000 × *g*, 4°C) and washed two times with 300 μl of SP lysis buffer containing 400 mM KAc and two times with 300 μl of SP lysis buffer containing 100 mM KAc (with centrifugation for 1 min at 2000 × *g* at 4°C between each step). The beads were then resuspended in SDS-PAGE sample buffer and heated for 10 min at 70°C before being loaded on a 12% acrylamide gel. The run was stopped as soon as the sample entered the separating gel so that all proteins from the sample were located within one single band. After Coomassie blue staining, the sample band was excised from the gel and analyzed by mass spectrometry.

### Mass spectrometry

Peptides for mass spectrometry analysis were generated by in-gel digestion for 1 h at 50°C in the presence of 20 mM ammonium bicarbonate, 0.01% ProteaseMax (Promega), and 24 ng sequencing-grade trypsin (Promega) according to the manufacturer's instructions. DDA (data-dependent acquisition) spectra were acquired using a Synapt G2si instrument (Waters) in the positive ion mode using the continuum data format and lock mass calibration. In the MS mode, spectra were acquired over the range of *m*/*z* = 350–2000 excluding the mass window 421–422 (trypsin autodigest), and in the MS/MS mode the spectra acquisition was performed over the range 50–2000 using charge state recognition of ions with two and three charges and eight MSMS channels. In both the MS and MS/MS mode, the scan time was 0.4 s and the interscan time was 0.015 s. The setting for the cone voltage was 40 V. Fragmentation in the MS/MS mode was performed using MSTrap collision energy profiles ranging from 20 to 25 V in the low mass range and from 30 to 45 V in the high mass range. Spectra were acquired from 10 to 100 min. Nano liquid chromatography separation of peptides was performed at a flow rate of 276 nl/min at 35°C using a combination of a Trap V/M Symmetry C18 column (100 Å, 5 μm, 180 μm × 20 mm) (Waters) and an Acquity UPLC M-Class peptide/BEH C18 analytical column (130 Å, 1.7 μm, 75 μm × 250 mm) (Waters). Solvent A was water with 0.1% formic acid. Solvent B was 75% acetonitrile, 25% isopropanol and 0.1% formic acid, and the gradient was as follows: 0.5 min, 5% B; 1 min 5% B; 74 min, 41% B; 82 min, 95% B; 106 min 95% B; 114 min, 5% B. Processing of the DDA data was performed using the Protein LynxGlobal server 3.0 software (Waters) using the default settings for FAST DDA data, including lock spray calibration and fast deiosotoping for the MS and MS/MS mode. Database searches using the peak lists of the processed mass spectra were performed using the Mascot search engine (version 2.6) in the UniprotKB/Swissprot database (version 2017_01) with a taxonomy filter for *S. pombe* sequences and in the Mascot database of contaminants. The search parameters permitted a mass error of 5 ppm for MS mode and 0.05 Da for MS/MS mode. Modifications included fixed modification of cysteine residues by carbamidomethylation, variable oxidation of methionine, and variable deamidation of asparagine and glutamine. The data were deposited at the PRIDE partner repository with the dataset identifiers PXD020907 (for pull-downs using oligonucleotides with polyA tails) and PXD020921 (for pull-downs using oligonucleotides without polyA overhangs).

### PhenDC_3_ sensitivity assay

PhenDC_3_ (cat# SML2298, Sigma-Aldrich) was prepared in DMSO. The *leu1-32 ade6-M216 oga1::kanR* (54) or *ded1-1D5 leu1-32* (55) strains (kind gifts from Prof. Hirofumi Aiba and Prof. Beáta Grallert, respectively) were mated with the *ade6-M210 bfr1::hygr pmd1::natr* strain (SAK27 strain; kind gift from Prof. Tarun Kapoor) (56) to generate the YKY37-YKY40 strains. The cells were grown in PMG media at 30°C until a density of 10^6^ cells/ml before adding 50 μM PhenDC_3_. The culture was then grown for another 12 h at 30°C for the *oga1Δ* strains (YKY39, YKY40) or at 35°C for the *ded1-1D5* strains (YKY37, YKY38). A serial dilution of each culture was then spotted on PMG plates that were incubated for 2 days at 30°C.

Doubling times of WT (SAK27) and *oga1Δ* strains (YKY39, YKY40) were determined by inoculating 1 million cells/ml in EMM2 media containing 0.07% DMSO (v/v) or 50 μM PhenDC3. Cultures were grown at 30°C at 180 rpm and counted after 12 h using a Bürker chamber. Subsequently, the cells were diluted to 1 million cells/ml in the presence of fresh DMSO or 50 μM PhenDC_3_, grown for an additional 12 h, and counted to calculate the doubling times. Three independent experiments were performed to calculate the doubling time using the following formula: doubling time = *t*/log_2_ (*x*/*x*_0_), where *t* is time in hours, *x* is the number of cells at 12 h, and *x*_0_ is the number of cells at 0 h.

### Purification of recombinant proteins

cDNAs of *oga1*, *dbp2*, and *ded1* were cloned into the pET24d vector for overexpression of histidine-tagged recombinant proteins in *Escherichia coli* Rosetta 2 cells (MERCK). The cells were grown at 37°C in 1 L of LB containing 50 μg/mL kanamycin and 34 μg/mL chloramphenicol until an OD of 0.4–0.6. IPTG was added to a final concentration 0.05 mM, and the cells were grown for another 4 h at 30°C before harvesting by centrifugation (15 min, 8000 × *g*, 4°C, JLA8.1, Beckman Coulter). The cell pellet was resuspended in lysis buffer (25 mM Tris–HCl pH 7.5, 500 mM NaCl, 10% glycerol, 0.02% igepal and 10 mM imidazole) supplemented with 3 μL DNase I (ThermoFisher, CAT# 18047019), 2 mM β-mercaptoethanol and EDTA-free cOmplete™ protease inhibitors (Roche) and lysed with a cell disruptor (Constant Systems Ltd) at a pressure of 25 000 psi. The sample was centrifuged (30 min, 20 000 × *g*, 4°C, JA25.50, Beckman Coulter), and the supernatant was incubated (1 h, 4°C) with 2 mL cOmplete™ His-Tag purification resin (MERCK) previously equilibrated in lysis buffer. The mixture was then poured into a gravity flow column and the resin was washed with 30 mL of wash buffer (same composition as the lysis buffer but with 20 mM imidazole). The elution was performed stepwise with buffer containing 200, 400 and 600 mM imidazole. The eluates were analyzed by SDS-PAGE, pooled together according to their purity, and desalted in 50 mM Tris pH 7.5, 100 mM NaCl, 10% glycerol and 1 mM DTT using a PD-10 desalting column (GE Healthcare). Protein variants were generated by the QuickChange Lightning Site-Directed Mutagenesis Kit (Agilent Technologies) and purified similarly as wild-type proteins.

### Electrophoretic mobility shift assay (EMSA) and helicase assay

The oligonucleotides were end labeled using T4 kinase (ThermoScientific) and γ-ATP at 37°C and then purified on native polyacrylamide gels containing 10 mM NaCl (for RNA substrates) or KCl (for DNA substrates). EMSA was carried out in 20 μL reaction mixtures containing 0.4 nM ^32^P-radiolabeled DNA/RNA, 50 mM Tris, 50 mM NaCl (for RNA substrates) or KCl (for DNA substrates), 2 mM MgCl_2_, 2 mM DTT, 0.25 mg/ml BSA, and various concentrations of Oga1/Dbp2/Ded1 protein variants or mitochondrial single-stranded DNA binding protein (mtSSB). The mixture was incubated for 10 min at 30°C before adding 4 μL of 6× loading buffer (40% glycerol, 0.2% bromophenol blue, 60 mM EDTA). The samples were separated by electrophoresis on a 10% native acrylamide gel in Tris/Borate/EDTA buffer. The gel was then dried on Whatman paper, exposed overnight to a phosphorimager (Fujifilm), visualized with a Typhoon 9400 Variable Mode Imager (GE Healthcare), and quantified with the ImageQuant software. The helicase assay was performed similarly to EMSA with the exception that 1 mM ATP was included in the reaction mixture. The reaction was stopped by adding 4 μl of 6× helicase stop buffer (40% glycerol, 0.2% bromophenol blue, 60 mM EDTA, 0.6% SDS and 2 mg/mL proteinase K) before a further incubation of 10 min at 37°C.

### ATPase assay

Reaction samples (50 μL) containing 50 mM Tris–HCl pH 8.5, 100 mM KCl, 5 mM MgCl_2_, 2 mM DTT, 1 mM ATP, 200 nM oligonucleotide and 20 nM Dbp2 or 60 nM Ded1 were incubated for 90 min at 30°C in a Nunc MaxiSorp flat-bottom 96-well plate (Affymetrix eBioscience). A total of 100 μL of BIOMOL Green reagent (Enzo Life Sciences) was added to the reaction followed by a 25 min incubation at room temperature before measuring the absorbance at 620 nm using an Infinite M200 plate reader (TECAN). The amount of phosphate released was calculated using the phosphate standard provided in the kit.

### G4 unfolding trap assay

The G4 oligonucleotide substrates at a final concentration of 0.2 nM (RNA-2G-G4, DNA-2G-G4) or 0.4 nM (5′-RNA-G4, DNA-3G-G4) were prepared in 50 mM Tris–HCl pH 7, 50 mM KCl (for DNA) or 50 mM NaCl (for RNA), 2 mM MgCl_2_, 2 mM DTT, 0.25 mg/mL BSA and 0 or 1 mM ATP. The reaction was started by adding 40 nM protein or peptide (RGGNYRRGGYGRGGFRRGG (RGG) or AGGNYRAGGYGAGGFRAGG (AGG)) and 1 nM trap oligonucleotide (RNA-2G-G4), 0.2 nM trap oligonucleotide (DNA-2G-G4), or 0.4 nM trap oligonucleotide (5′-RNA-G4 or DNA-3G-G4) and incubated at 30°C for 20 min. At regular time intervals 10 μL of the reaction mixture was removed and immediately quenched with 10 μL stop buffer (40% glycerol, 0.2% bromophenol blue, 60 mM EDTA, 0.6% SDS, 1 μM anti-trap oligonucleotide). The samples were then run for 140 min at 110 V in a 20% acrylamide gel containing 10 mM KCl for DNA or 10 mM NaCl for RNA. The gel was then dried and exposed to a phosphorimager, visualized on a Typhoon 9400 Variable Mode Imager (GE Healthcare), and quantified with the ImageQuant software. Controls were performed by using proteins boiled for 10 min in 0.1% SDS or by adding 2 nM PhenDC_3_ to the reaction mixture.

## RESULTS

### Affinity purification of *S. pombe* G4-binding proteins

In order to affinity purify G4-binding proteins, we used a well-characterized stable G4 structure from the *S. pombe* rDNA sequence that pauses DNA replication *in vitro* and is bound and unwound by the Pfh1 DNA helicase (Table 1) (47–48,15,57–58). The rDNA oligonucleotide was folded in KCl and showed a typical parallel G4 structure, as depicted by the characteristic circular dichroism (CD) spectrum that featured a negative peak at 245 nm and a positive peak at 264 nm (Supplementary Figure S1A). As non-G4 controls, we used both a G-rich scrambled sequence (scr) and a mutated rDNA sequence (m4), where one guanine from each G-tract was substituted with a thymine (Table 1). The scr sequence did not display a typical G4 structure, as the positive peak was shifted and broadened, and unlike the rDNA G4 sequence (Supplementary Figure S1A), it was unstable at temperatures of 45°C and higher (Supplementary Figure S1B). The m4 sequence displayed a spectrum consistent with single-stranded DNA (Supplementary Figure S1C-D). The experiments were carried out using the rDNA G4 sequence either with or without a poly(A)_10_ overhang on the 5′ end of the oligonucleotide. The presence of the poly(A)_10_ overhang does not influence the topology of the G4 structure (47).

**Figure 1.**
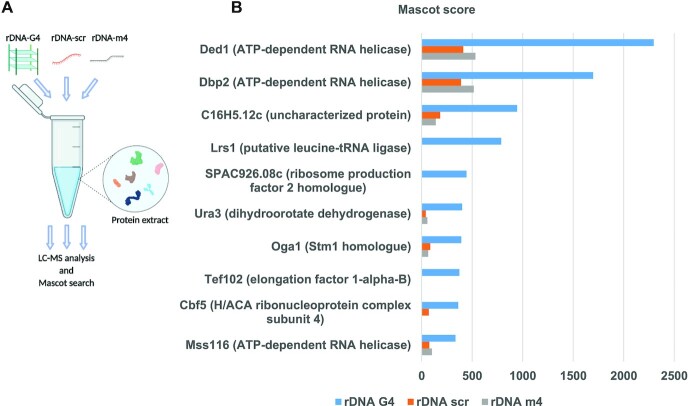
The identification of putative G4-binding proteins by mass spectrometry. (**A**) Graphical illustration of the experimental procedure. Affinity purification was performed with three different biotinylated oligonucleotides—rDNA-G4, rDNA-scr and rDNA-m4—mixed with either *S. pombe* whole protein cell extract or nuclear protein extract. LC–MS analysis and the Mascot search engine were used to identify the protein hits from each separate experiment. Image was created with BioRender.com. (**B**) The top 10 hits are presented based on the average Mascot score of three different affinity purification experiments with G4 oligonucleotides compared to the non-G4 controls (rDNA-scr and rDNA-m4).

To affinity purify G4-binding proteins, we incubated the different DNA templates with whole-cell lysates or nuclear-enriched protein extracts isolated from wild type (WT) *S. pombe* cells and performed liquid chromatography–mass spectrometry (LC–MS) analysis (Figure 1A). From the LC-MS analysis, we identified 30 putative G4-binding proteins with a Mascot score of 100 or higher and that also showed a higher Mascot score for the rDNA G4 structure pull-downs compared to the non-G4 controls (Supplementary Table S2). By performing a gene ontology (GO) search with these proteins using the PANTHER classification system (59), we found, for instance, enrichment for nucleic acid binding (19 genes), RNA helicase activity (five genes), and translation regulator activity (seven genes) (Supplementary Table S3). The majority of the proteins were found in all three different pull-downs (Supplementary Table S2), but some were only detected in the nuclear-enriched extracts. For instance, the rRNA-processing protein Ebp2, the splicing factor U2AF subunit Prp2 and the rRNA processing protein Mis3 were all only found in the nuclear-enriched extracts (Supplementary Table S2). Peptide motif searches using the *S. pombe* resource PomBase (60) revealed that 8 of the 30 hits had putative RGG motifs in their amino acid sequences (Supplementary Table S2). Among the top 10 hits, we found three DEAD box RNA helicases (Ded1, Dbp2, and Mss116), one protein involved in metabolic pathways (ura3), two involved in ribosome biogenesis (SPAC926.08c, Cbf5), two involved in translation elongation (Tef102, Lrs1), one involved in Target of rapamycin (TOR) signaling (Oga1), and one uncharacterized protein (C16H5.12c) (Figure 1B, and Supplementary Table S2). For this study, we focused our in-depth characterization on three of the hits that possessed putative RGG motifs, namely the RNA helicases Ded1 (human ortholog DDX3) and Dbp2 (human ortholog DDX5) and the *S. cerevisiae* Stm1 homologue Oga1, a yeast-specific protein.

### *In vivo* characterization of affinity-purified proteins

**Figure 2.**
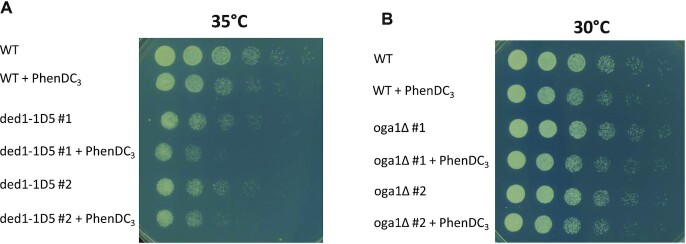
*In vivo* experiments in the presence of a G4 stabilizing compound. (**A**) The temperature-sensitive strain *ded1-1D5* was grown in the presence or absence of the G4 stabilizer PhenDC_3_. Two clones of the strain (denoted #1 and #2) (Supplementary Table S4) were tested, serial-diluted (five-fold dilutions), and spotted on a PMG plate. (**B**) The *oga1Δ* strain was tested and spotted as in (A).

*S. pombe* cells are multi-drug resistant due to efficient efflux pumps (56). However, *S. pombe* mutant cells missing the two genes encoding ABC transporter proteins Pmd1 and Bfr1 are sensitive to many drugs (56), including the bisquinolinium phenanthroline G4 stabilizer PhenDC_3_ (51) and a quinazoline-based G4-stabilizing ligand (15). In order to assess the role of the hit proteins in cells upon G4 stabilization, we performed spot dilution assays with untreated and PhenDC_3_-treated cells. The *ded1^+^* and *dbp2^+^* genes are both essential, and therefore we were unable to perform spot dilution assays with strains in which these genes were deleted. However, a temperature-sensitive mutant *ded1-1D5* was described previously (55). To test if *ded1-1D5* cells are sensitive to G4 stabilization, we mated *pmd1Δ bfr1Δ* with *ded1-1D5*, in which the Ded1 protein is depleted when the cells are grown at restrictive temperatures (55). The *ded1-1D5 pmd1Δ bfr1Δ* (hereafter referred to as *ded1-1D5*) mutant strain showed growth defects compared to the *pmd1Δ bfr1Δ* (hereafter referred to WT) strain when grown at 35°C. Treatment with PhenDC_3_ enhanced this growth defect in the *ded1-1D5* mutant compared to WT cells. (Figure 2A), suggesting that there might be an additive effect in *ded1-1D5* PhenDC_3_-treated cells. Due to clumping of the *ded1-1D5* cells, we were unable to determine their doubling time. We also assessed the growth of an *oga1Δ pmd1Δ bfr1Δ* strain (hereafter referred to *oga1Δ*) in the presence of PhenDC_3_ and compared this to WT cells. The growth of both PhenDC_3_-treated WT and PhenDC_3_-treated *oga1*Δ cells was affected (Figure 2B), and the doubling times of PhenDC_3_-treated WT and PhenDC_3_-treated *oga1Δ* cells were 180 and 184 minutes while non-treated WT and *oga1Δ* cells showed doubling times 175 and 173 min, respectively (Supplementary Figure S2). However, no significant growth differences were found between the *oga1Δ* and the WT cells (Figure 2B, Supplementary Figure S2), indicating that G4 stabilization in the *oga1Δ* mutants does not cause severe problems for the cells.

### Recombinant Oga1, Dbp2 and Ded1 bind to G4 DNA *in vitro*

**Figure 3.**
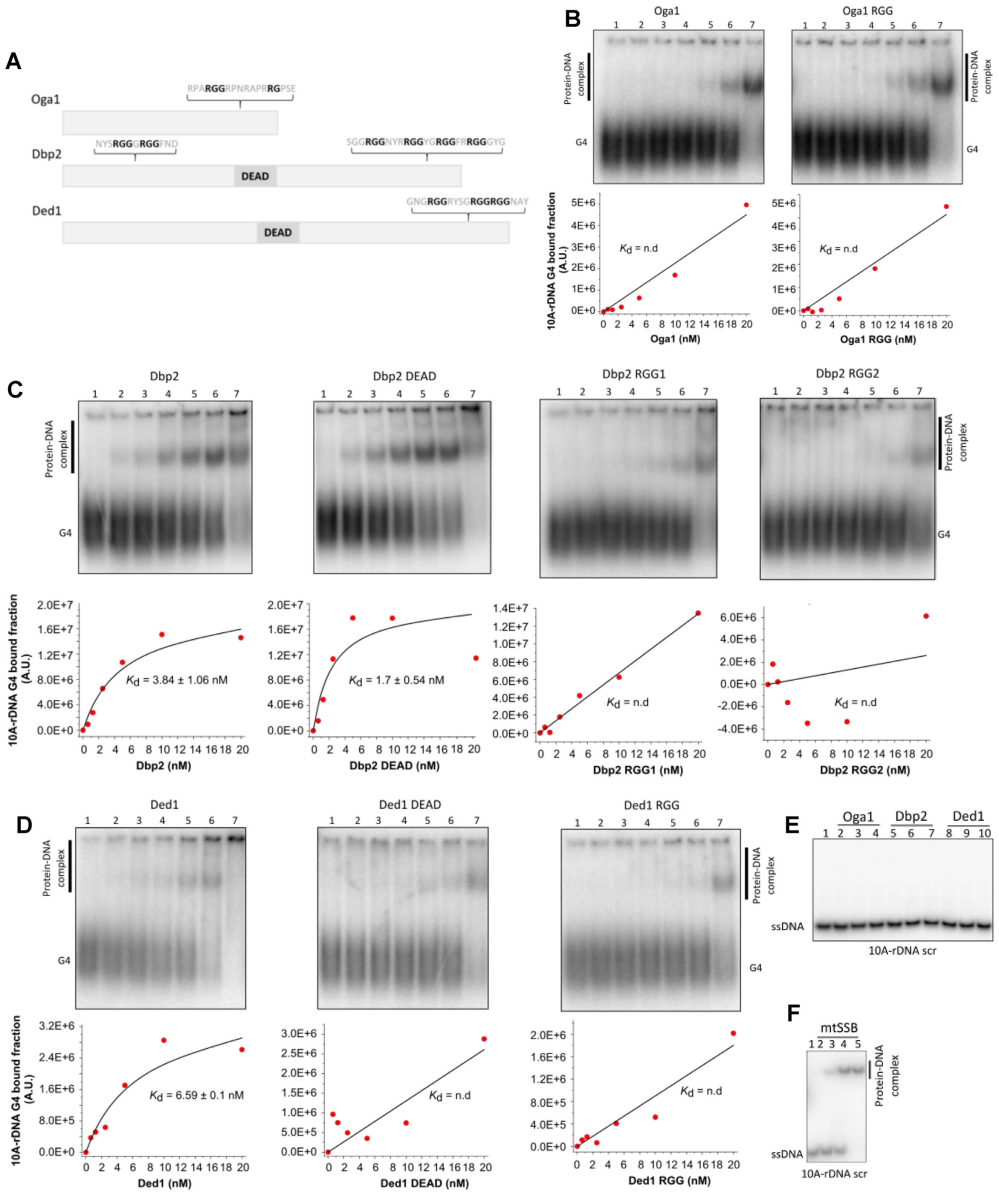
Oga1, Dbp2, and Ded1 bind to G4 DNA *in vitro* but not to single-stranded DNA. (**A**) Schematic view of Oga1, Dbp2, and Ded1 proteins showing the location of the putative RGG domains and DEAD box domain. EMSA performed on 10A-rDNA G4 with increasing concentrations of Oga1 variants (**B**), Dbp2 variants (**C**), Ded1 variants (**D**). Lane 1 (no protein); lane 2 (0.625 nM protein); lane 3 (1.25 nM protein); lane 4 (2.5 nM protein); lane 5 (5 nM protein); lane 6 (10 nM protein); lane 7 (20 nM protein). Black lines correspond to a 1:1 fitting model. Data fitting with the 1:1 binding model was obtained with Bindfit by using multiple global fitting methods (Nelder–Mead method) (92,93). The *k* values show the mean of two independent experiments ± SD., and n.d. indicates not possible to determine. (**E**) EMSA performed on the 10A-rDNA scr oligonucleotide with increasing concentrations of Oga1, Dbp2 and Ded1. The proteins were used at concentrations of 0.1, 1 and 10 nM. Lane 1 does not contain any protein. (**F**) EMSA performed on the 10A-rDNA scr oligonucleotide with increasing concentrations of mtSSB. The protein was used at concentrations of 0.01, 0.1, 1 nM and 10 nM. Lane 1 does not contain any protein.

Next, we determined the *in vitro* G4 binding properties of the three proteins (Figure 3). We first expressed and purified recombinant Oga1, Dbp2 and Ded1 using a C-terminal poly-histidine tag construct in *E. coli* (Supplementary Figure S3A) and then used EMSA to assess the binding affinities of the purified proteins to the rDNA G4 with a poly(A)_10_ overhang (10A-rDNA G4), the same *S. pombe* rDNA G4 that was used as the bait in our pull-down experiments (Figure 1). We observed protein concentration-dependent shifts of the G4 oligonucleotide band for all three proteins, indicating that Oga1, Dbp2, and Ded1 directly bind G4 DNA (Figure 3B–D). Both Dbp2 and Ded1 bound the G4 DNA with very high affinity exhibiting dissociation constants (*K*_d_) of 3.84 ± 1.06 nM and 6.59 ± 0.1 nM, respectively (Figure 3C, D). However, Oga1 bound the G4 DNA with low affinity, and we were unable to determine the *K*_d_ of Oga1 for the G4 substrate under our experimental conditions (Figure 3B). In sharp contrast, none of the proteins bound to the single-stranded DNA sequence 10A-rDNA scr (Figure 3E). As expected, the mtSSB showed binding to the single-stranded 10A-rDNA scr substrate (Figure 3F). These data suggest that all three proteins selectively bind G4 DNA.

### RGG motifs in Oga1, Dbp2 and Ded1

To further understand the G4-binding properties of these proteins, we next examined the putative RGG motifs in the sequences of the three proteins (Figure 3A), as 8 of our 30 G4-associated hits contained this motif. Ded1 possessed a typical RGG domain in the C-terminal region consisting of three RGG repeats separated by zero or four amino acid residues and with a tyrosine residue in the spacer region between two of the RGG repeats (Figure 3A). Dbp2 had two putative RGG domains, one located in the N-terminal region encompassing two RGG repeats (RGG1) and the other located in the C-terminal region, with four RGG repeats separated by either two or three amino acids (RGG2) (Figure 3A). Also, an aromatic amino acid residue was located in between each RGG2 repeat in Dbp2. Oga1 contained a domain that is reminiscent of an RGG motif in the C-terminal region, and this domain included one RGG and one RG repeat, but they were separated by six amino acid residues (Figure 3A).

For each putative RGG motif, we generated a protein variant where we substituted each arginine by an alanine (hereafter referred to as RGG mutants) (Supplementary Figure S3A, S3B). For the DEAD-box helicases (Dbp2 and Ded1), we also created helicase-inactive variants by substituting the catalytic glutamic acid from the Asp-Glu-Ala-Asp (DEAD) box with a glutamine (DEAD mutants; Ded1 E337Q and Dbp2 E277Q) (Supplementary Figures S3A, S3B). All protein variants were recombinantly expressed and purified to near homogeneity (Supplementary Figure S3A).

### The RGG motifs in Ded1 and Dbp2 are necessary for G4 DNA binding

To test the G4 binding activity of the protein variants, we again performed EMSA using 10A-rDNA G4 (Figure 3). We did not observe any difference in the binding of Oga1 RGG compared to WT for the G4 structure, suggesting that the arginine residues in the putative RGG domain are not important for Oga1’s binding to the G4 DNA (Figure 3B).

Similar to WT Dbp2, the helicase-inactive variant, Dbp2 DEAD, fully retained its ability to bind 10A-rDNA G4 with a *K*_d_ of 1.7 ± 0.54 nM, whereas Dbp2 RGG1 displayed weaker binding as detected by the decrease in the intensity of the bands corresponding to the protein-DNA complex (Figure 3C). Dbp2 RGG2 showed the weakest binding.

Ded1 DEAD bound to rDNA G4 weaker than WT Ded1, while Ded1 RGG showed the weakest binding compared to WT Ded1 and Ded1 DEAD (Figure 3D). Due to the weak binding of Dbp2 RGG1, Dbp2 RGG2, Ded1 DEAD and Ded1 RGG to the G4 substrate, we were not able to determine their *K*_d_ values under our experimental conditions.

Furthermore, we tested the G4 binding activity of the protein variants using other highly stable G4 DNA structures, the intermolecular parallel human Z33 G4 DNA from the *SupF* gene (Supplementary Figure S1E) (61), and an intramolecular G4 DNA from the human *c-MYC* promoter region (c-MYC G4) (Supplementary Figures S1F, S3, Table 2). Again, no difference in binding to these two substrates was detected by the Oga1 RGG compared to WT (Supplementary Figure S3C–E). The EMSA with Z33 G4 DNA and c-MYC G4 DNA also demonstrated weaker binding of the Ded1 and Dbp2 RGG variants compared to their WT counterparts, which confirmed that the RGG domains of Dbp2 and Ded1 are important for binding to G4 DNA (Supplementary Figure S3C, S3D, S3F and S3G). We conclude that the RGG motifs in the C-terminus of Dbp2 and Ded1 are important for G4 DNA binding.

We also tested the binding of all the protein variants to a single-stranded RNA (ssRNA) oligonucleotide and to two parallel G4 RNA structures with either 5′ poly A overhangs (10A-G4 RNA) or 5′ and 3′ poly U overhangs (5U-G4-5U RNA) (Supplementary Figure S1G, Table 2). We again performed EMSA and found that all protein variants bound to the ssRNA and G4 RNA structures (Supplementary Figure S4). WT Oga1 had the weakest affinity to ssRNA among the tested proteins (Supplementary Figure S4). In contrast to the G4 DNA binding, some of the RGG variants bound more strongly than the WT proteins to both the ssRNA and G4 RNA substrates (Supplementary Figure S4). Also, we did not observe any clear trends in the binding affinities of the different proteins to G4 RNA structures. These results may perhaps be explained by the fact that our RNA G4 oligonucleotides not only encompass the G4 structure, but also have ssRNA overhangs either on the 5′ end or on both the 5′ and 3′ ends. Due to these limitations of our RNA binding assays, the role of the RGG motif of the tested proteins to RNA G4s was difficult to interpret. However, we can conclude that all WT proteins bind to G4 RNA structures.

Finally, in all subsequent work we only used the Dbp2 RGG2 variant with mutations in the C-terminus that showed the most deleterious effect for G4 DNA binding, and we will refer to this as Dbp2 RGG throughout the rest of the manuscript.

### The Dbp2 and Ded1 WT and RGG variants are catalytically active

Although the superfamily 2 DEAD-box RNA helicases show diverse mechanistic activities, a common property of these helicases is that the presence of ssRNA stimulates their ATPase activity (62). We therefore determined the ATPase activity of the different DEAD-box protein variants. In the presence of ssRNA, both Dbp2 and Ded1 were able to hydrolyze ATP faster than in the absence of ssRNA, showing that ssRNA stimulates their ATPase activity (Supplementary Figure S5A, Table 2). As expected, the ATPase-inactive variants Dbp2 DEAD and Ded1 DEAD lost their ability to hydrolyze ATP (Supplementary Figure S5A). The Dbp2 RGG and Ded1 RGG retained their ATPase activity in the presence of ssRNA (Supplementary Figure S5A), thus demonstrating that although the RGG variants were impaired in their ability to bind G4 DNA structures, they were still catalytically active in the presence of ssRNA.

**Figure 4.**
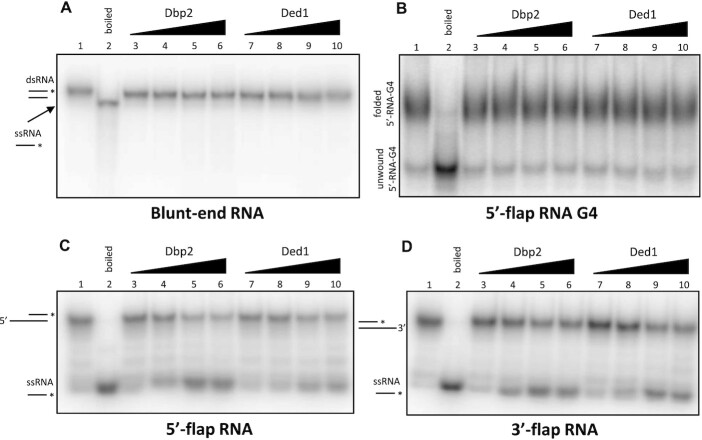
Dbp2 and Ded1 unwind double-stranded RNAs encompassing either a 5′ or 3′ flap. Helicase assay performed on (**A**) blunt-end double-stranded RNA, (**B**) 5′-flap RNA G4 (5′-RNA-G4), (**C**) 5′-flap RNA (5′-RNA) and (**D**) 3′-flap RNA (3′-RNA) substrates. The proteins were used at concentrations of 1, 10, 50 and 100 nM.

We next determined their ability to unwind an intermolecular G4 RNA structure containing three stacks of G-tetrads and a 5′ poly(A)_10_ overhang (10A-RNA-3G-G4) and other RNA substrates (Table 2). We first tested whether WT Dbp2 and Ded1 could unwind the blunt-ended double-stranded RNA and the 10A-RNA-3G-G4 substrate, but neither of the proteins had the ability to unwind these structures (Figure 4A, B). However, as also demonstrated in the ATPase assay, both proteins were active because they were able to unwind partial duplex RNA oligonucleotide substrates encompassing either a 5′ or 3′ ssRNA overhang sequence (Figure 4C, D). These results also confirmed that the purified WT helicases were active, but we could not detect unwinding of the G4 RNA structures under the conditions used in these experiments.

### Binding of Dbp2 and Ded1 unfolds G4 DNA and RNA structures in an ATP-independent manner

**Figure 5.**
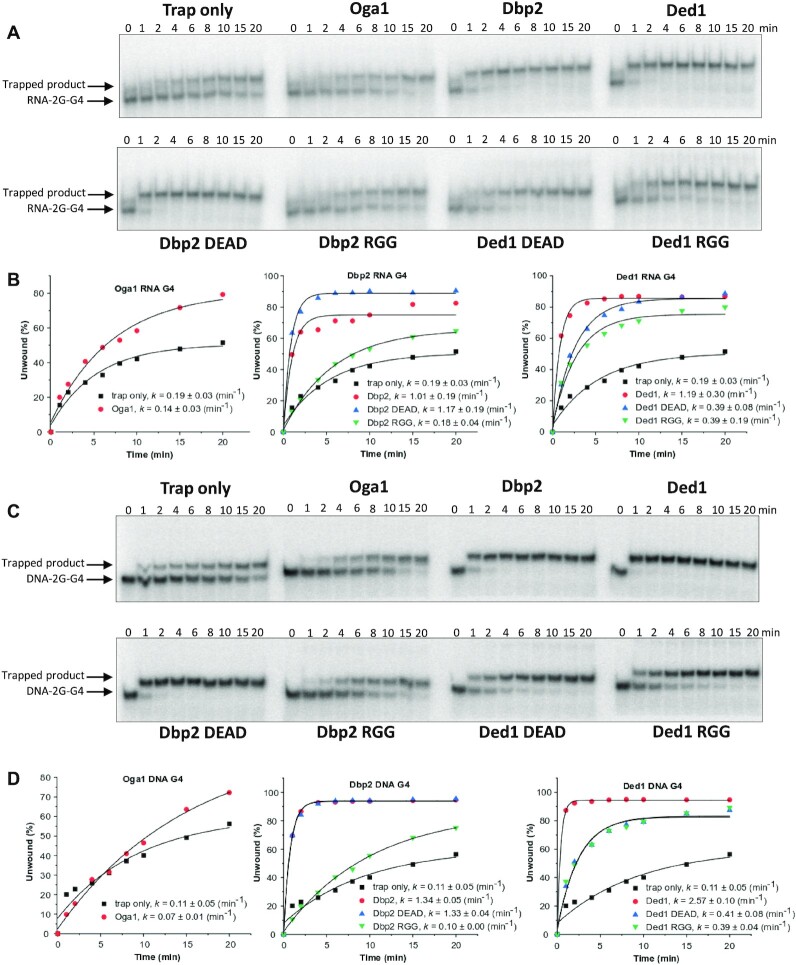
Dbp2 and Ded1 destabilize RNA and DNA G4 made up of two G-tetrad stacks. (**A**) The G4 RNA helicase assay was performed in the presence of a trap oligo complementary to the G4 RNA sequence. An aliquot of the reaction mixture at different time intervals up to 20 min was then loaded on a gel. (**B**) The amount of unwound G4 RNA was quantified in three different experiments, and the average amount was fitted to a monoexponential function. The *k* values show the mean of three independent experiments ± SD. (**C**) The G4 DNA helicase assay was performed as in A). (**D**) The amount of unwound G4 DNA was quantified in two different experiments, and the average amount was fitted to a monoexponential function. The *k* values show the mean of two independent experiments ± SD.

Next, we performed a trap-assisted G4 unfolding assay to determine if the G4 unwinding was undetected because of rapid refolding of the G4 substrate. In this assay, a trap oligonucleotide complementary to the G4 sequence that prevents the unfolded G4 structure from refolding was included (28). Dbp2 and Ded1 were again unable to unfold the stable 10A-RNA-3G-G4 substrate (Supplementary Figure S5B), suggesting that these proteins cannot unwind highly stable G4 structures. In general, many DEAD-box RNA helicases are non-processive helicases that unwind short dsRNA molecules with low thermostability, and their unwinding efficiency is reduced on substrates with high stability (63). Unimolecular G4 structures with two G-tetrad stacks can also form (64), and these are commonly less stable than G4 structures with three stacks. We therefore again performed the trap-assisted G4 unfolding assay but with a less thermostable G4 RNA structure that had two stacks of G-tetrads (RNA-2G-G4) (28) instead of the one tested in Figure 4B that had three stacks of G-tetrads (10A-RNA-3G-G4). We found that in this experimental setting, the trap oligonucleotide itself was able to unfold the RNA-2G-G4 structure, and we observed increased amounts of the trapped unfolded product (upper band) with increased incubation time (Figure 5A, B). Adding either Dbp2 or Ded1 in the reaction mixture resulted in a more than five-fold faster rate of G4 RNA destabilization with rate constants (*k*) of 1.01 ± 0.19 and 1.19 ± 0.30 min^−1^, respectively, compared to the trap-only sample with a *k* of 0.19 ± 0.03 min^−1^ (Figure 5A, B). However, addition of Oga1 did not accelerate the rate of G4 RNA destabilization (*k* = 0.14 ± 0.03 min^−1^). We also performed this assay in the absence of ATP and found that destabilization of the G4 RNA structure by Ded1 and Dbp2 was ATP-independent (Supplementary Figure S6).

### The RGG mutated variants have reduced G4 unfolding activity

The helicase assays described above suggested that the efficient G4 RNA destabilization activity was not due to the helicase activity of the DEAD-family helicases but was perhaps due to the binding of the proteins to the G4 structure. To test this, we repeated the helicase trap assay in the presence of the different protein variants. These results showed that the helicase-inactive Dbp2 DEAD unfolded the G4 RNA structure with a *k* of 1.17 ± 0.19 min^−1^, which was similar to WT Dbp2 thus confirming that the G4 RNA destabilization by Dbp2 does not require ATP hydrolysis (Figure 5A, B). In contrast, the Dbp2 RGG variant did not induce G4 unfolding (*k* = 0.18 ± 0.04 min^−1^) showing a similar destabilization trend as the trap-only samples (Figure 5A, B). Thus, the binding of Dbp2 is sufficient to destabilize the G4 structure thus causing it to unfold.

The ATPase hydrolysis-inactive Ded1 DEAD and Ded1 RGG showed about a three-fold slower destabilization effect (*k* = 0.39 ± 0.08 min^−1^) on the G4 RNA structure compared to WT Ded1 (*k* = 1.19 ± 0.30 min^−1^) (Figure 5A, B), indicating that the destabilization of the G4 RNA by Ded1 DEAD and Ded1 RGG was impaired compared to WT Ded1 (Figure 5A, B). These data also suggest that in addition to arginines other residues in the RGG domain of Ded1 might be important for the G4 interaction.

We also carried out these trap helicase experiments using an equivalent DNA oligonucleotide, DNA-2G-G4, which similar to the RNA-2G-G4 had two G-tetrad stacks and which folded into a parallel G4 structure (Supplementary Figure S1H), as well as a more stable DNA G4 structure composed of three stacks of G-tetrads (DNA-3G-G4) (28). None of the proteins were capable of unfolding the stable DNA-3G-G4 substrate (Supplementary Figure S5C). In contrast, Dbp2 and Ded1 were able to very efficiently destabilize the DNA-2G-G4 substrate showing a *k* of 1.34 ± 0.05 min^−1^ and *k* of 2.57 ± 0.10 min^−1^, respectively. Complete destabilization was reached after 2 min incubation, while the trap-only control had a *k* of 0.11 ± 0.05 min^−1^ and showed about 20% destabilization after the same incubation time (Figure 5C, D). The rate of G4 DNA destabilization was faster than the G4 RNA destabilization for both helicases (Figure 5), perhaps because G4 DNA is generally less thermostable than G4 RNA (65). Oga1 (*k* = 0.07 ± 0.01 min^−1^) showed a very similar trend as the trap-only control, indicating that Oga1 could not destabilize the G4 DNA structure (Figure 5C, D). Unfolding of the G4 DNA was also observed with Dbp2 DEAD (*k* = 1.33 ± 0.04 min^−1^), but not with Dbp2 RGG (*k* = 0.10 ± 0.00 min^−1^). Although no differences were observed between Ded1 DEAD (*k* = 0.41 ± 0.08 min^−1^) and Ded1 RGG (*k* = 0.39 ± 0.04 min^−1^) (Figure 5C, D), both protein variants were in fact much slower than WT Ded1. Therefore, ATP hydrolysis may be more important for G4 DNA destabilization for Ded1 compared to Dbp2. However, WT Ded1 was still very efficient in G4 DNA destabilization in the absence of ATP (Supplementary Figure S6C, S6D). In fact, we did not detect any significant change in the global unfolding pattern of these protein variants in the absence of ATP (Supplementary Figure S6C, S6D). Finally, to test if the RGG domain alone is enough for G4 destabilization we performed helicase assays with the Dbp2RGG WT and mutant (AGG) peptides using DNA-2G-G4. However, neither peptides were able to destabilize the DNA-2G-G4 substrate (Supplementary Figure S7A, S7B). Taken together, these results show that both Dbp2 and Ded1 are able to destabilize less stable DNA G4 structures and that this ability is dependent on the RGG domain when it is folded in a larger polypeptide chain, such as in the full-length protein.

### Stabilization of G4 DNA and RNA by the G4 stabilizer PhenDC_3_ impairs unfolding by Ded1 and Dbp2

Next, we carried out the helicase trap assay experiments using the G4 stabilizer PhenDC_3_ to determine how the stability of the G4 structures affects the unfolding activity. The use of PhenDC_3_ in the helicase trap assay resulted in reduced unfolding of RNA-2G-G4 by the different proteins, but no quantification could be performed because the gel bands were not well-defined (Supplementary Figure S8A). These results also confirm what has been shown with other DEAD-box helicases where the thermostability of the substrates affects the destabilization effect of the DEAD-box helicases (63) and are in agreement with our observations with the three G-tetrad G4 structures (Figure 4B, Supplementary Figure S5B and S5C).

PhenDC_3_ also impaired the unfolding of DNA-2G-G4 by the proteins (Supplementary Figure S8B and S8C). Dbp2 and Dbp2 DEAD were still able to unfold DNA-2G-G4 (*k* = 0.13 min^−1^), but at much slower rates than in the absence of G4 stabilization (Figure 5C, D, Supplementary Figure S8B and S8C). Unfolding of DNA-2G-G4 by Dbp2 RGG was even more reduced, showing that the RGG motif is important for the G4-unfolding activity of Dbp2. The presence of PhenDC_3_ slowed down the rate of unfolding more for Ded1 RGG (*k* determination was not possible) and Ded1 DEAD (*k* determination was not possible) compared to WT Ded1 (*k* = 0.16 min^−1^) (Supplementary Figure S8B and S8C).

Furthermore, we performed a control experiment where all of the proteins were denatured by boiling the protein samples in SDS. The denatured proteins lost their ability to unfold RNA-2G-G4 and DNA-2G-G4 structures (Supplementary Figure S9). These results suggest that G4-binding proteins favor the unfolding of less thermostable G4 RNA and DNA structures by a mechanism that is dependent on a properly folded protein.

### Dbp2 RGG is necessary for G4 RNA destabilization

**Figure 6.**
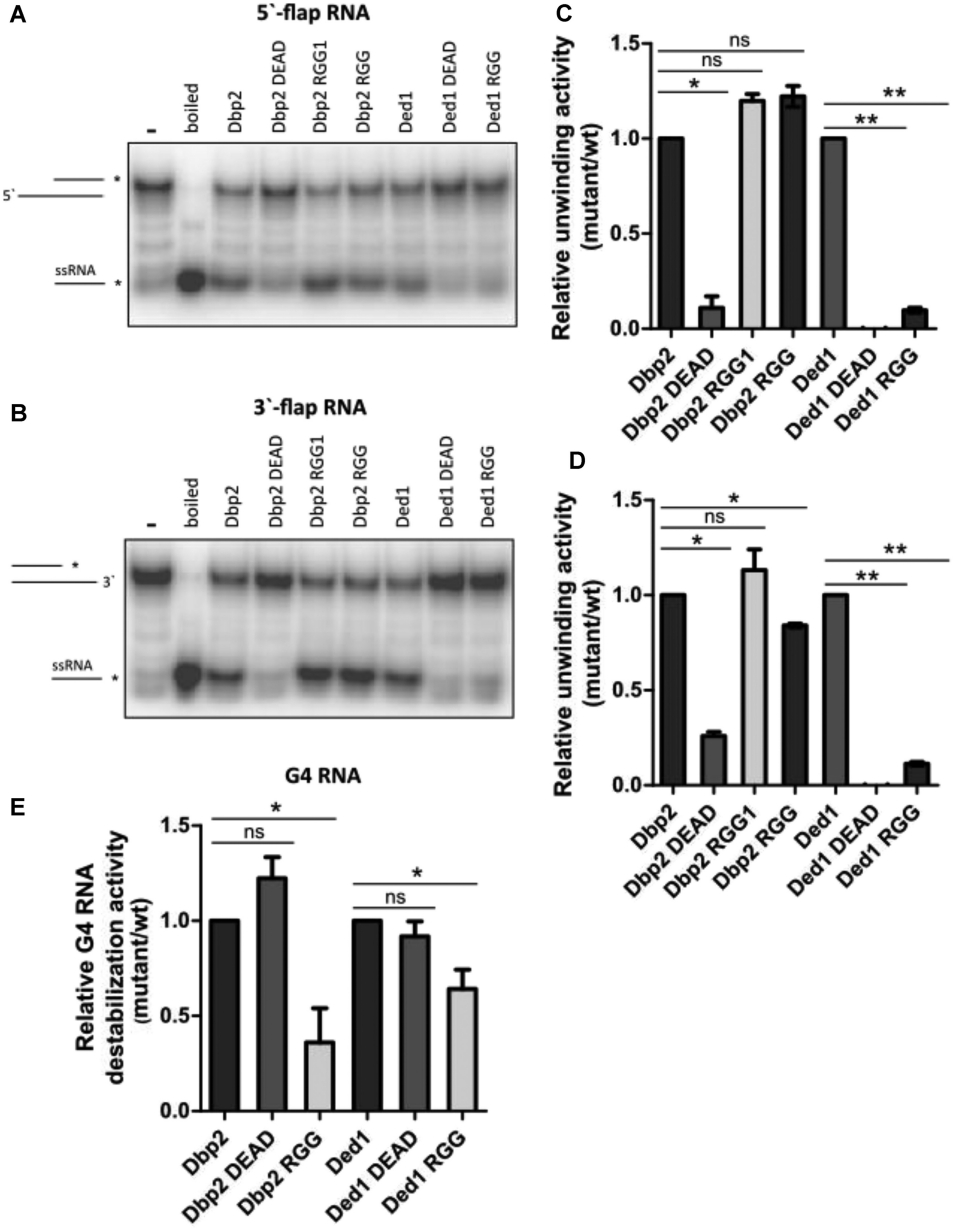
The RGG domain of Dbp2 specifically contributes to the destabilization of RNA G4 made up of two G-tetrad stacks. Helicase assay performed on (**A**) 5′-flap RNA (5′-RNA) and (**B**) 3′-flap RNA (3′-RNA) substrates using 10 nM Dbp2 and Ded1 variants. Quantification of relative unwinding of (**C**) 5′-flap RNA (5′-RNA), and (**D**) 3′-flap RNA (3′-RNA) substrates. Relative unwinding activity was determined by normalizing the amounts of unwound products by the protein variants to the unwound product of WT protein. (**E**) Quantification of the relative destabilizing activity of RNA-2G-G4. The amount of unwound products at the 10 min time point from Figure 5A was used to determine the relative G4 destabilization by subtracting the amount of unwound product of the trap only from those of the proteins and then normalizing the amounts of unwound products of the protein variant to the unwound product of WT protein. Statistical analysis was performed using Student's *t*-test. *P* < 0.05 and ** *P* < 0.01; ns non-significant.

We also determined the ability of Dbp2 and Ded1 variants to unwind the partial duplex RNA oligonucleotide substrates flanking either a 5′ or 3′ ssRNA overhang sequence and compared this non-G4 unwinding activity with the G4 destabilization activity of the RNA-2G-G4 substrate (Figure 6). As shown above (Figure 4C and D), Dbp2 and Ded1 were able to unwind the partial duplex RNA oligonucleotide substrates flanked by either a 5′ or 3′ ssRNA overhang sequence. As expected, both Dbp2 DEAD and Ded1 DEAD did not unwind these non-G4 substrates (Figure 6A-D) because this unwinding activity requires ATP hydrolysis. However, both DEAD mutants destabilized the RNA-2G-G4 substrate (Figure 6E).

Finally, although the Dbp2 RGG mutants could not destabilize the RNA-2G-G4 substrate (Figures 5, 6E), they were active in unwinding the non-G4 substrates (Figure 6B, C). Ded1 RGG mutation reduced both the unwinding activity of the non-G4 substrates and RNA-2G-G4 destabilization (Figure 6). These results suggest that the G4 destabilization is localized to the RGG domain in Dbp2, while the RGG domain in Ded1 contributes to both non-G4 and G4 destabilization activities.

## DISCUSSION

Using a proteomics approach, we identified 30 putative G4 binding proteins from *S. pombe*. We selected three of them that possessed putative RGG domains—Oga1, Dbp2 and Ded1—and showed that they are all able to directly bind both DNA and RNA G4 structures *in vitro*. In *S. pombe*, only two other proteins, Pfh1 and Rif1, have so far been shown to directly associate with G4 structures. However, neither of these proteins were included in our top 30 hits that encompassed both cytosolic, nuclear, and mitochondrial proteins, most likely because neither of these two proteins are highly abundant in cells (66). Therefore, our approach captured G4 binding proteins that are available at high copy number in the cell, and most likely there are more G4 binding proteins in *S. pombe* waiting to be discovered.

Five of the 30 hits are annotated as helicases, including Ded1, Dbp2, Mss116, Ste13 and SPAC694.02, and they belong to the DEAD/DEAH-box RNA helicases. DEAD box helicases are involved in a wide range of cytoplasmic and nuclear processes, including transcription, ribosome biogenesis, pre-mRNA splicing, and protein translation (63). We found that both Ded1 and Dbp2 unfolded partially duplex RNA substrates independently of the orientation of the flap and that neither unfolded a blunt-end RNA substrate. These results are consistent with previous observations from other proteins from this helicase family and suggest that Dbp2 and Ded1 unwind RNA through a different mechanism than translocating helicases, most likely using a mechanism called local strand separation (67–69).

Furthermore, while we were determining the *in vitro* properties of these helicases, a study in *S. cerevisiae* was published that showed that *S. cerevisiae* Dbp2 (ScDbp2), Mss116 (ScMss116), and Ded1 (ScDed1) bind and unfold RNA G4s *in vitro* (28). Also, another study using human cells detected the human orthologs of Ded1 and Dbp2—DDX3 and DDX5—when taking an affinity proteomics approach (70). In addition to G4 binding, we also showed that both Dbp2 and Ded1 unfolded DNA G4 structures that were made up of two G-tetrad stacks. In the *S. cerevisiae* study (28), only G4 DNA structures that were made up of three G-tetrads were tested. Similar to *S. pombe* Ded1 and Dbp2, the *S. cerevisiae* homologs were unable to unfold such G4 DNA structures (Supplementary Figure S5C), thus suggesting that the G4 unfolding activity of Dbp2 and Ded1 is correlated with the thermostability of the G4 structures. In fact, the presence of PhenDC_3_ significantly slows down the destabilization effect by Ded1 and Dbp2. This correlation is also observed with the human DEAH-box helicase DHX36 (RHAU), and DHX36 is also unable to efficiently unfold more thermostable G4 structures or G4 structures that are stabilized by G4-stabilizing ligands (71). In contrast, FRET assays showed that DDX5 can unfold the thermostable *c-MYC* G4 DNA *in vitro* (72). Together, these studies demonstrate that the G4 binding/unfolding functions of these helicases are evolutionarily conserved. Based on our observations, we propose that Ded1 and Dbp2 use a non-processive mechanism to destabilize G4 DNA and RNA molecules and that less thermostable structures are more readily unfolded. Alternatively, their enzymatic activity may be enhanced by other proteins that facilitate binding and/or destabilization of G4 DNA and/or RNA structures. For instance, subunits of *S. cerevisiae* eIF4F interact with ScDed1 and stimulate both its *in vitro* helicase (73) activity and *in vivo* functions (74).

The Oga1 protein is conserved in fungi and has a homologue in *S. cerevisiae* called Stm1 (ScStm1). ScStm1 is a telomere and ribosome-associated protein that binds to G4s and acts as a translation modulator under nutrient stress conditions (75–78). Earlier, Oga1 was identified as a protein involved in the longevity of the fission yeast, and by using whole cell protein extracts it was shown that Oga1 binds to G4 structures; however, it was not shown if the binding was direct or if it was an indirect binding through interaction of Oga1 with other G4 binding proteins (54). In this study, we used purified Oga1 protein and showed that Oga1 directly bound to both DNA G4 and RNA G4.

The RGG motif has been identified as a key protein feature for G4 binding proteins such as fragile X mental retardation 1 (FMRP) (79), TLS/FUS (80), EWS (81), CIRBP (46), nucleolin (82,83), and DDX3 (84). In addition to G4-recognition, the RGG motif has been found in both G4-stabilizing and destabilizing proteins. Here, we first showed that mutations in the RGG motifs in Dbp2 and Ded1 impair the proteins’ G4 DNA interactions, providing additional evidences that Ded1 and Dbp2 directly interact with G4 DNA structures. We also showed that the RGG motif is important for both G4 DNA and RNA destabilization. The putative RGG motif in Oga1 is not a ‘classic’ RGG domain, and our mutational biochemical studies also supported that this region may not be important for G4 DNA binding. At least the arginine residues in the putative RGG motif that were mutated to alanine (R249A and R259A) did not play a significant role in G4 DNA binding. Other protein hits from our affinity purification experiments that possessed a putative RGG motif were Ste13, Sum2, Moe1, SPAC12G12.07c and Mss116. Mss116 and the uncharacterized protein SPAC12G12.07c were among our top 10 protein hits, and similar to Ded1 and Dbp2 they showed putative RG/G motifs in their C-terminal regions. SPAC12G12.07c is a conserved cytosolic fungal protein, but so far its functions are unknown. Mss116 has not been studied in *S. pombe*, but ScMss116 is important for mitochondrial splicing (85).

We also showed that unfolding of the G4 structures by Dbp2 and Ded1 does not require ATP, which is in accordance to results for ScDed1, ScDbp2 (28), and human DDX5 (72). This is in contrast to what is observed for translocating helicases, and this might be because the DEAD-box helicases lack processivity and have different mechanisms of nucleic acid unwinding (62) (see discussion above). The ATP hydrolysis independence of Dbp2 was further supported by using the catalytically inactive variant of Dbp2. For Ded1, these results were not fully supported and are in need of more detailed investigation. However, the loss of the G4 destabilization activity by the Ded1 RGG and Dbp2 RGG variants, which both were impaired in G4 DNA binding, suggests that Ded1 and Dbp2 are able to destabilize G4 structures simply by binding to these structures and that the binding is partly mediated/facilitated by the arginine residues in the RGG motif. In addition, the RGG motif in Dbp2 only contributes to G4 destabilization and not to RNA partial duplex unwinding, while the RGG domain in Ded1 contributes to both non-G4 and G4 destabilization activities.

Finally, although it is clear that Ded1 binds to G4 structures *in vitro*, our *in vivo* analyses in the presence of the G4 stabilizing compound PhenDC_3_ only showed a modest growth defect compared to untreated cells. Perhaps the small effect on cell growth is because other G4 binding/destabilizing proteins can compensate for Ded1 or because the depletion levels of Ded1 were not drastic enough to show more enhanced growth defects. Mutations in the Ded1 human ortholog DDX3 as well as dysregulated DDX3 expression are associated to several cancer types (86). Previous studies on the functions of Ded1 DEAD box helicase subfamilies suggest that one of the roles of this subfamily is in translation initiation, possibly by resolving 5′-UTRs. Similar to human cells, predicted G4 structures are enriched at 5′-UTRs in *S. pombe* cells (36). Therefore, unfolding G4 structures at 5′-UTRs might be one of *S. pombe* Ded1’s functions in regulating translation. In fact, WT DDX3 is associated with RNA G4 structure-containing 5′-UTR transcripts, and this association is reduced in RGG-mutated DDX3 (70). Together, these studies support the notion that unfolding G4 structures at 5′-UTRs might be one of the functions of the Ded1/DDX3 DEAD-box subfamily and perhaps one of the dysregulated functions in cancer cells (55,86–89). About 60% of the *S. pombe* Ded1 sequence is identical to ScDed1, and ScDed1 but not ScDbp2 can complement the growth of *S. pombe ded1-1D5* (55). Therefore, the cellular function of ScDed1 and *S. pombe* Ded1 seem to be complementary. Work on the Dbp2 DEAD box subfamilies suggests roles in RNA remodeling and transcription regulation (90,91). Similar to DDX3, the human Dbp2 ortholog DDX5 is connected to cancer predisposition (90), showing the importance of studying these G4 binding proteins at the molecular level.

## DATA AVAILABILITY

The mass spectrometry proteomics data have been deposited to the ProteomeXchange Consortium via the PRIDE partner repository with the dataset identifiers PXD020907 and PXD020921.

## Supplementary Material

gkab620_Supplemental_File
