# Supplementary material for: The RGG domain in the C-terminus of the DEAD box helicases Dbp2 and Ded1 is necessary for G-quadruplex destabilization
Source: Nucleic Acids Res. 2021 Jul 24;49(14):8339–54. doi: 10.1093/nar/gkab620 (PMC8373067; doi:10.1093/nar/gkab620)
Supplement: gkab620_Supplemental_File [file gkab620_supplemental_file.pdf]

Supporting data

The RGG domain in the C-terminus of the DEAD box helicases Dbp2 and Ded1 is necessary for G-quadruplex destabilization

Kevin Kok-Phen Yan, Ikenna Obi, and Nasim Sabouri*

Department of Medical Biochemistry and Biophysics, Umeå University, 901 87 Umeå, Sweden

*To whom correspondence should be addressed. E-mail: nasim.sabouri@umu.se

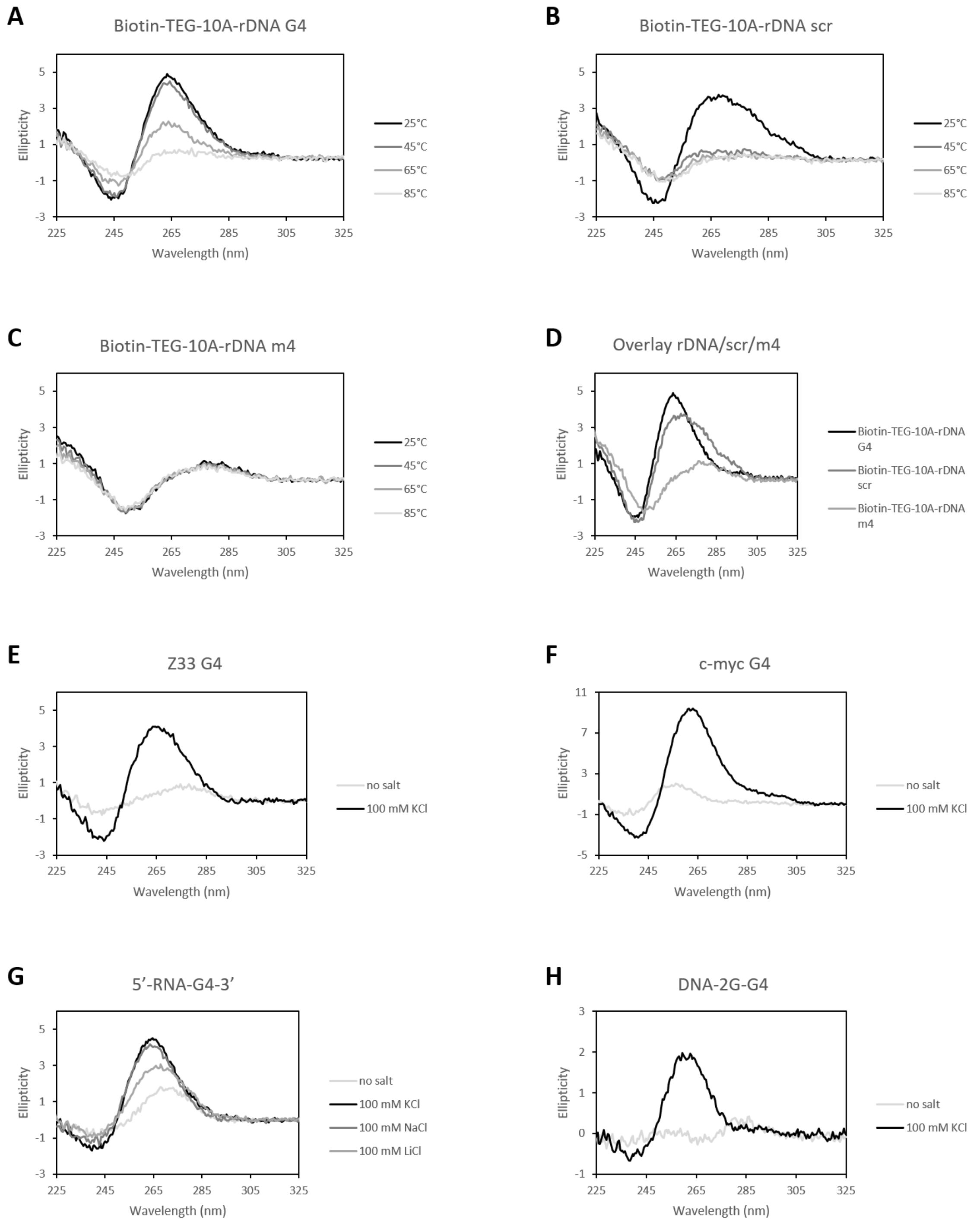

Figure S1. Formation of G4 structures by CD. CD spectra of **(A)** Biotin-TEG-10A-rDNA G4 DNA, **(B)** Biotin-TEG-10A-rDNA G4 DNA scr, and **(C)** Biotin-TEG-10A-rDNA G4 DNA m4 oligonucleotides. **(D)** Overlay of the CD spectra in A, B, and C. CD spectra of **(E)** Z33 G4 DNA, **(F)** *c*-MYC G4 DNA, **(G)** 5'-RNA-G4-3', and **(H)** DNA-2G-G4 oligonucleotides. If not otherwise indicated, the CD spectra were recorded at 25°C in either 0 mM or 100 mM KCl.

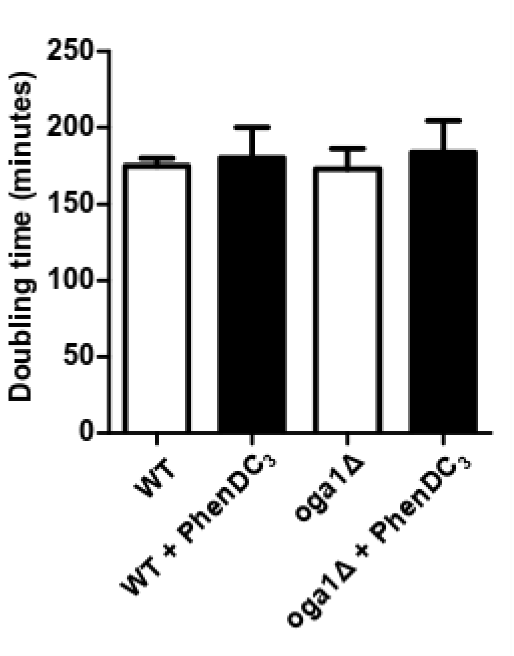

Figure S2. Exponentially growing WT and *oga1Δ* cells were grown in EMM2 media containing 0 (DMSO) or 50 µM PhenDC$_3$. Doubling times were determined for the cultures grown for 24h at 30°C. Three independent experiments were carried out. Error bars represent the standard deviation. The difference in growth was not statistically significant.

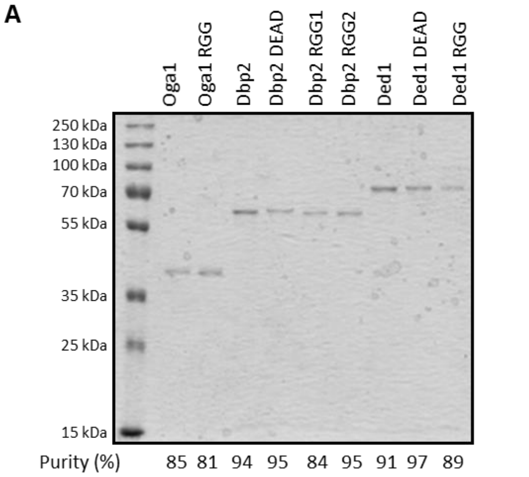

B

Mutant	Mutations
Oga1 RGG	R249A, R259A
Dbp2 DEAD	E277Q
Dbp2 RGG1	R35A, R39A
Dbp2 RGG2	R510A, R516A, R521A, R526A
Ded1 DEAD	E337Q
Ded1 RGG	R574A, R581A, R584A

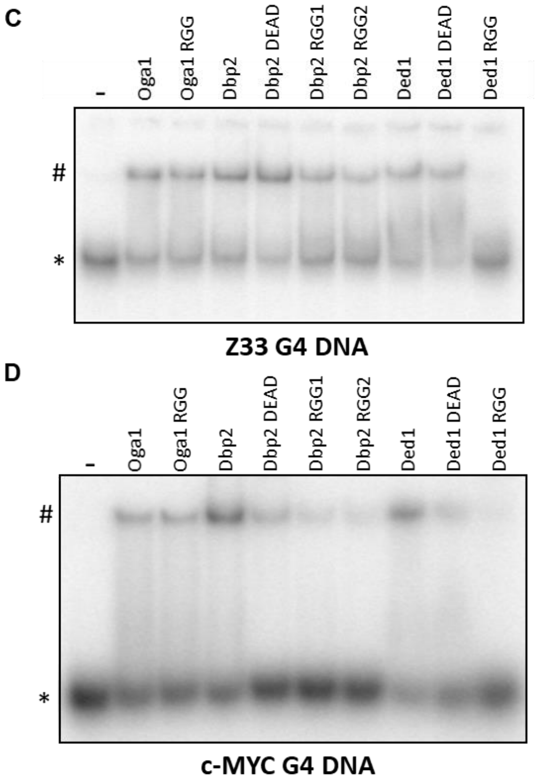

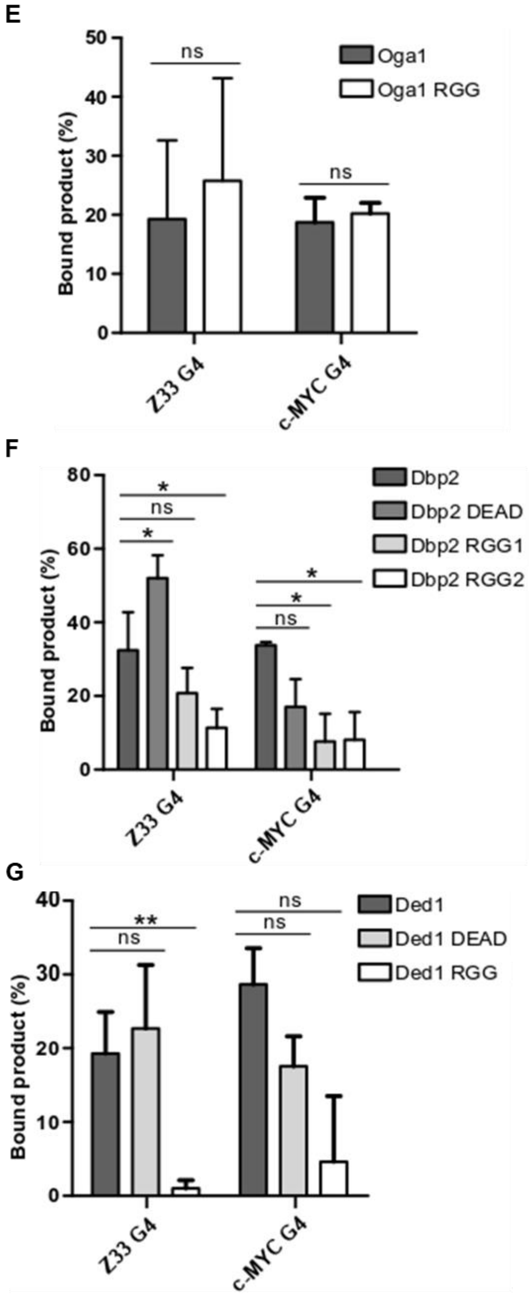

Figure S3. (**A**) Visualization of purified WT and mutated Oga1, Dbp2, and Ded1 proteins by SDS-PAGE. A total of 300 ng of each protein was loaded. (**B**) List of mutants generated for Oga1, Dbp2, and Ded1. Representative gel images for EMSA are shown. EMSA was performed with 10 nM of protein and 0.2 nM (**C**) Z33 G4 DNA, and **(D)** c-MYC G4 DNA substrates. The graphs represent quantification of the EMSA gels for the (**E**) Oga1 protein variants, (**F**) Dbp2 protein variants, and (**G**) Ded1 protein variants. The experiments were performed in duplicates with error bars showing the standard deviation. * and # indicate DNA and protein-DNA complex, respectively.

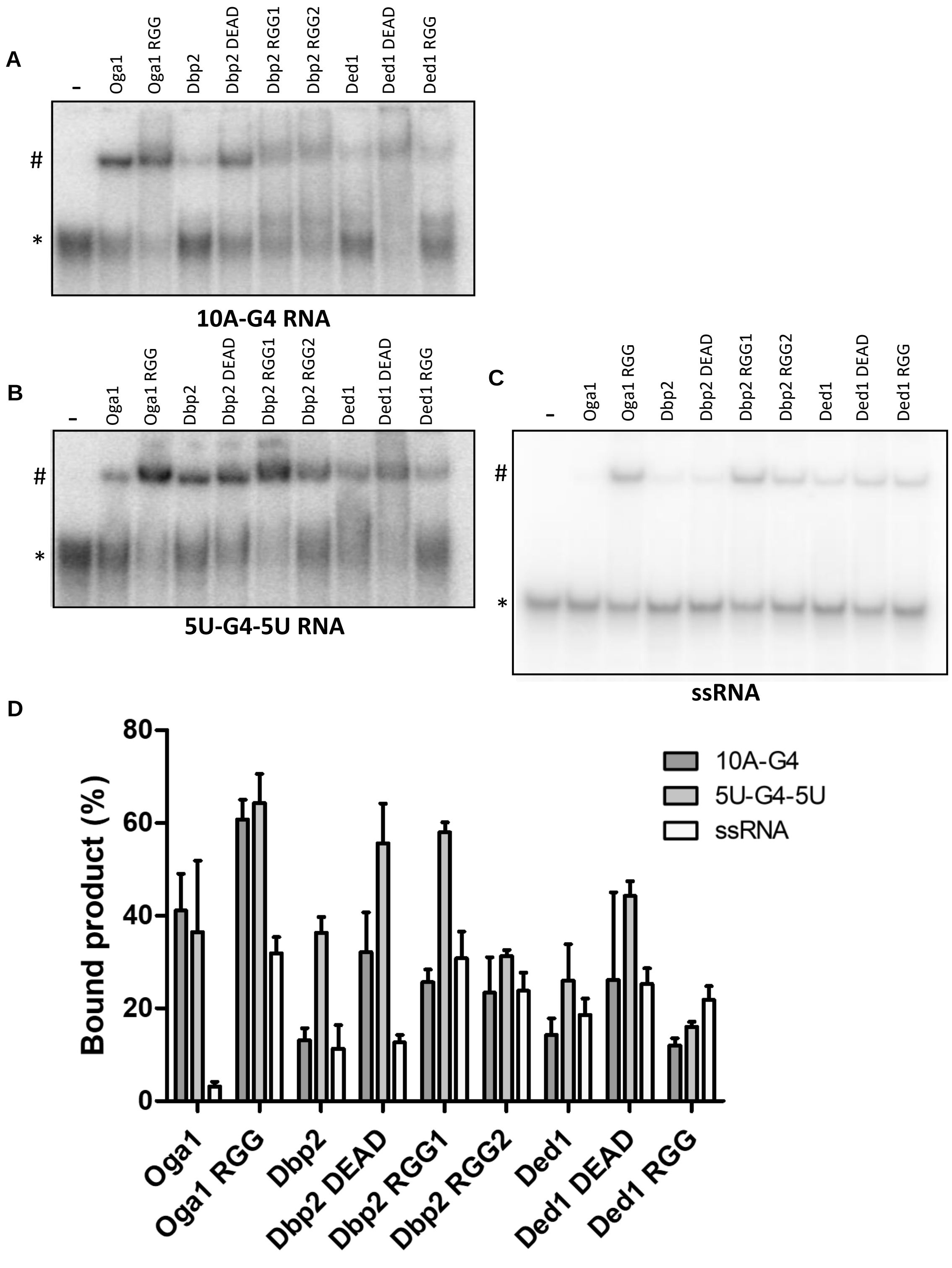

Figure S4. Effect of mutating the RGG domains on the binding of G4 RNA. EMSA was performed with 10 nM of protein and 0.2 nM **(A)** 5'-10A flap RNA-G4, **(B)** 5'-5U flap 3' 5U flap G4 RNA, and **(C)** ssRNA substrates. * and # indicate RNA and protein-RNA complex, respectively. **(D)** The quantification of RNA binding was performed in duplicate with error bars showing the standard deviation.

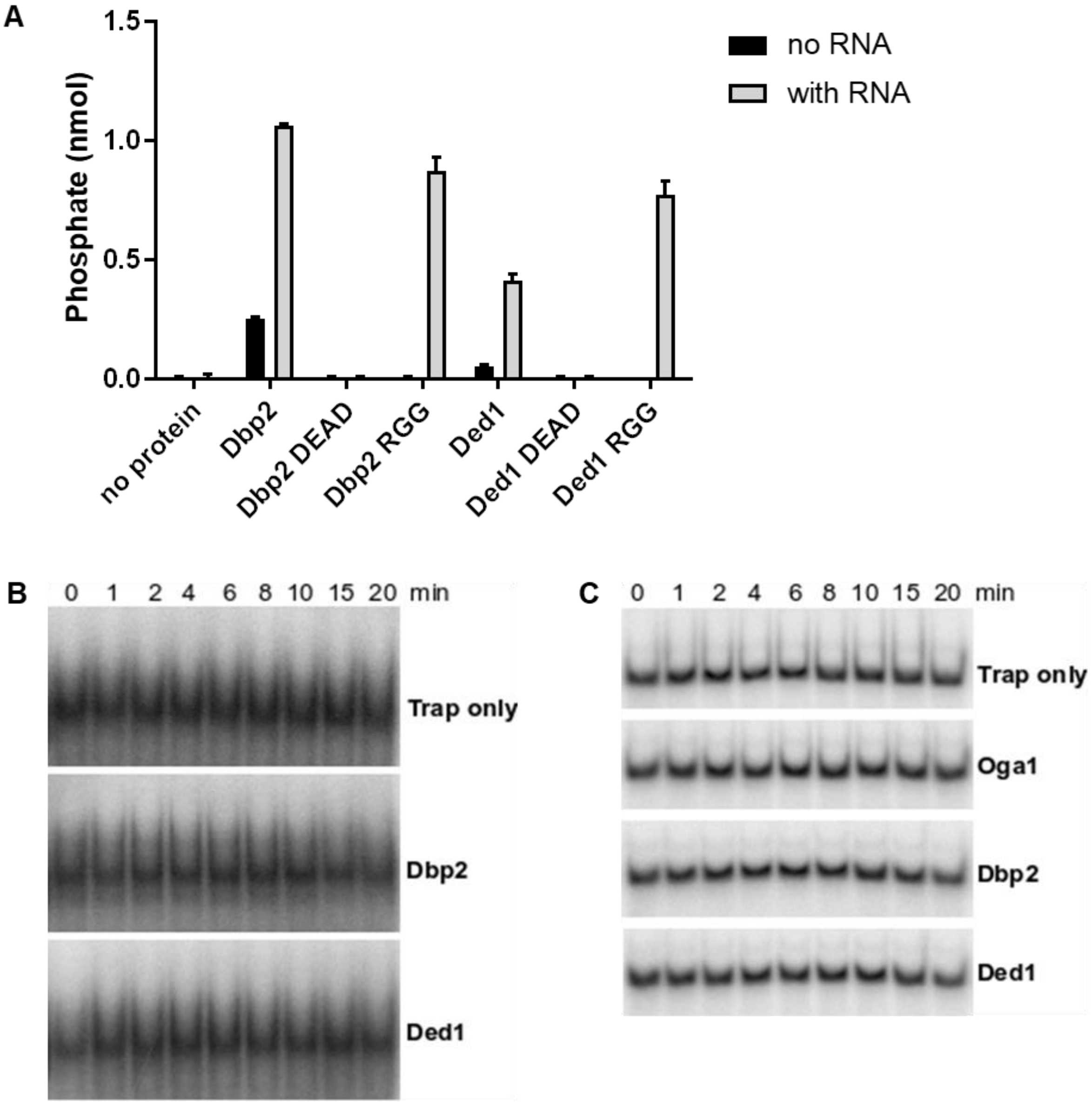

Figure S5. (**A**) RGG mutants but not DEAD mutants maintain their ATPase activity. The ATPase activity was measured in triplicate with 20 nM of Dbp2 protein or 60 nM of Ded1 protein with or without the presence of ssRNA oligonucleotides. The error bars represent the standard deviation. (**B**) Dbp2 and Ded1 are inactive in destabilizing 5′-flap RNA G4 (5′-RNA-G4). The G4 helicase assay was performed in the presence of a trap oligo complementary to the G4 RNA sequence. An aliquot of the reaction mixture at different time intervals up to 20 min was then loaded on a gel. (**C**) All variants are inactive in destabilizing DNA G4 made up of three G-tetrad stacks (DNA-3G-G4). The G4 DNA helicase assay was performed in the presence of a trap oligo complementary to the G4 DNA sequence. An aliquot of the reaction mixture at different time intervals up to 20 min was then loaded on a gel.

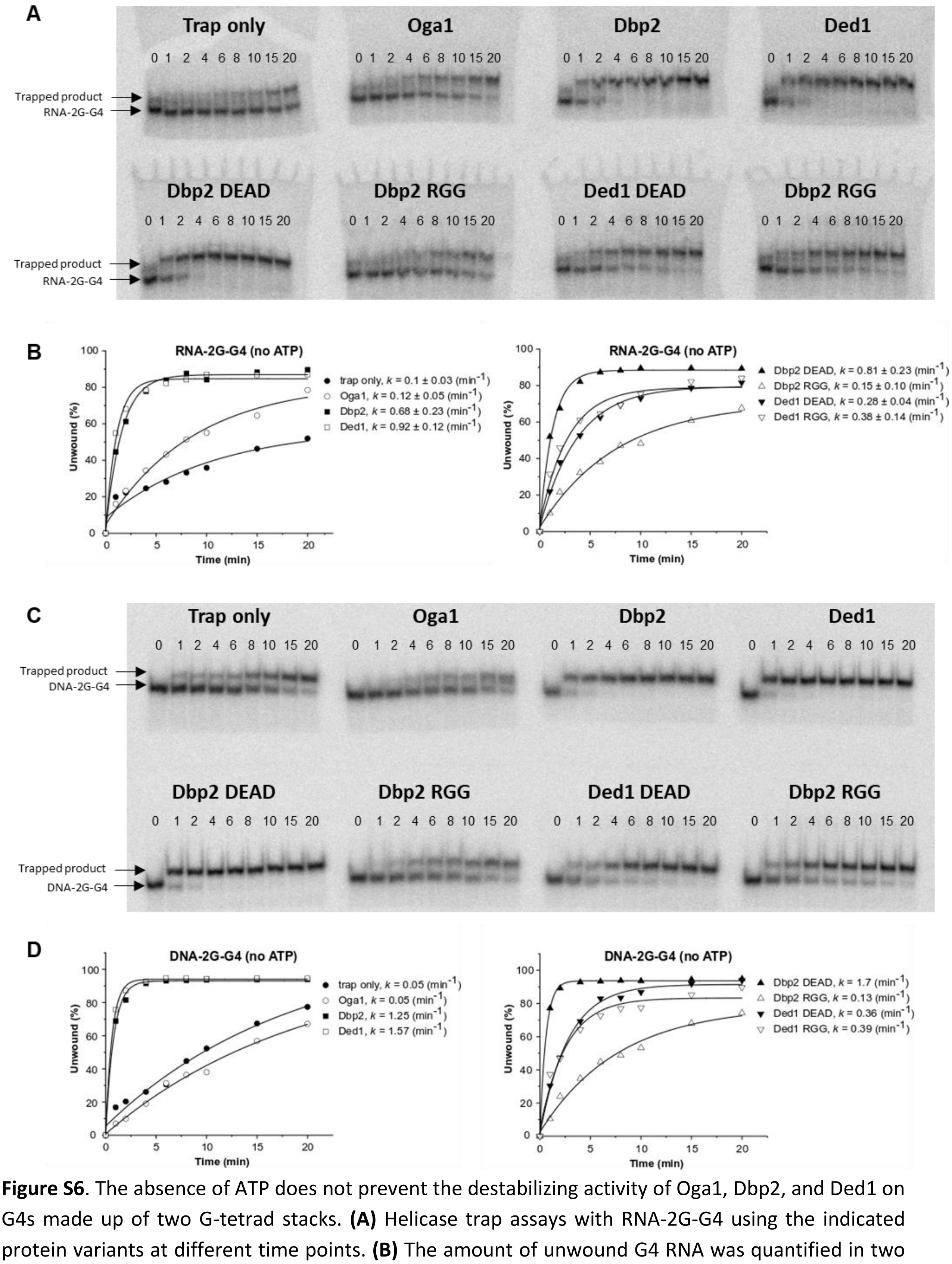

Figure S6. The absence of ATP does not prevent the destabilizing activity of Oga1, Dbp2, and Ded1 on G4s made up of two G-tetrad stacks. **(A)** Helicase trap assays with RNA-2G-G4 using the indicated protein variants at different time points. **(B)** The amount of unwound G4 RNA was quantified in two different experiments, and the average amount was fitted to a monoexponential function. *k* values show mean of two independent experiments ± SD. **(C)** Helicase trap assays on DNA-2G-G4 using the indicated protein variants at different time points. **(D)** The amount of unwound G4 DNA based on the gels in C) was quantified and fitted to a monoexponential function to determine *k* values.

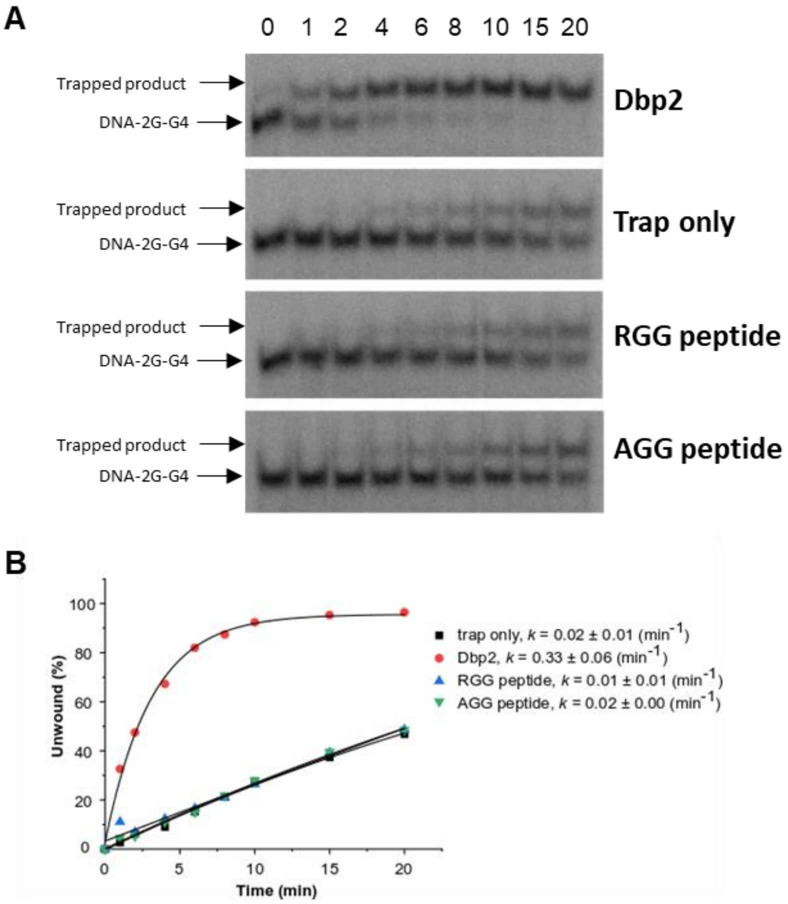

Figure S7. Dbp2 RGG peptide does not destabilize DNA G4 made up of two G-tetrad stacks. **(A)** The G4 DNA helicase assay was performed in the presence of a trap oligo complementary to the G4 DNA (DNA-2G-G4) sequence. An aliquot of the reaction mixture at different time intervals up to 20 min was then loaded on a gel. (**B**) The amount of unwound G4 DNA was quantified in two different experiments, and the average amount was fitted to a monoexponential function. *k* values show mean of two independent experiments ± SD.

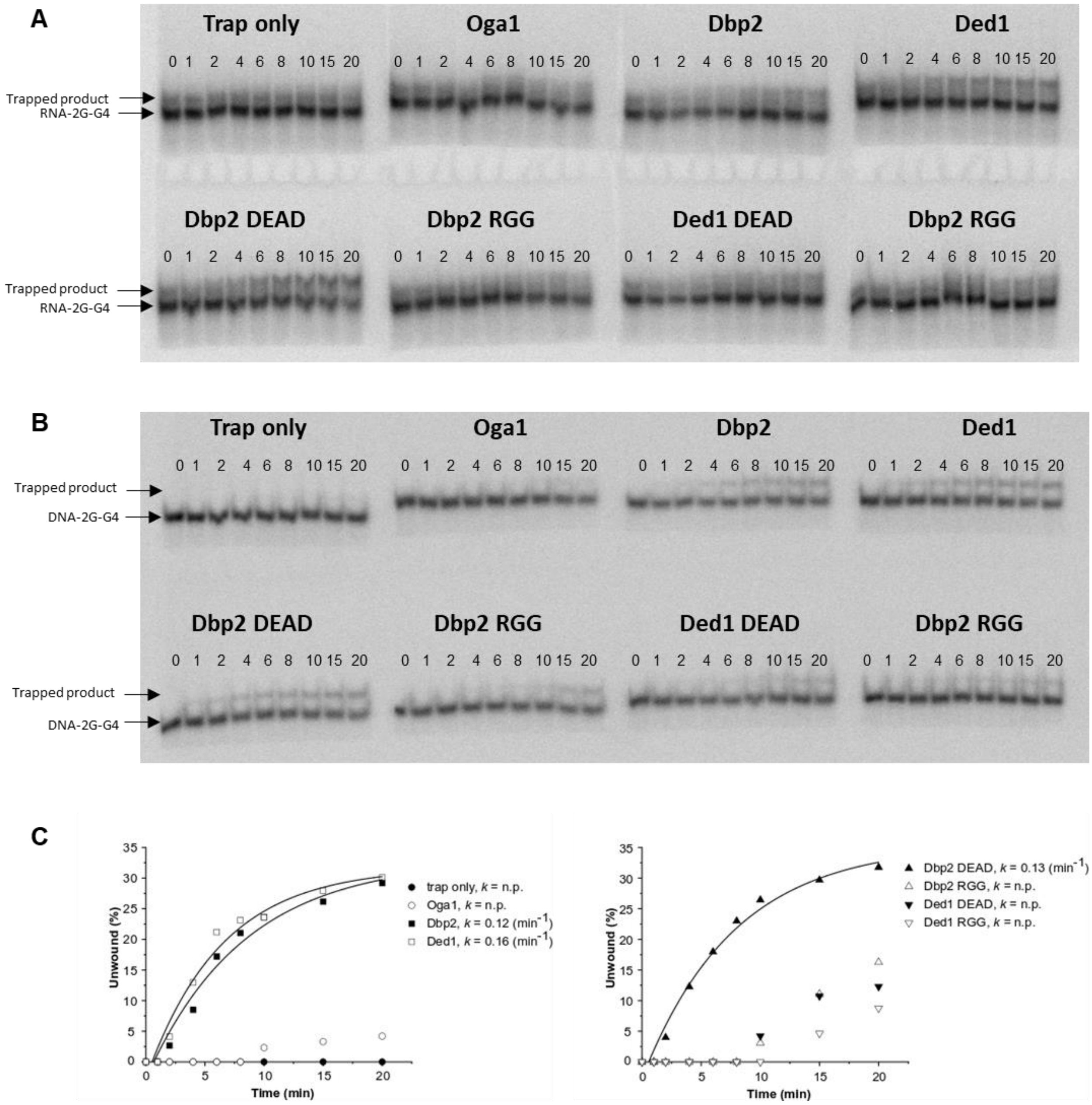

Figure S8. The presence of $PhenDC_3$ reduces the rate of the G4-destabilizing effect of Oga1, Dbp2, and Ded1. Helicase trap assays with **(A)** RNA-2G-G4 and **(B)** DNA-2G-G4 using the indicated protein variants at different time points. **(C)** The amount of unwound G4 DNA based on the gels in B) was quantified and fitted to a monoexponential function to determine *k* values. n.p. indicates not possible to determine the *k*.

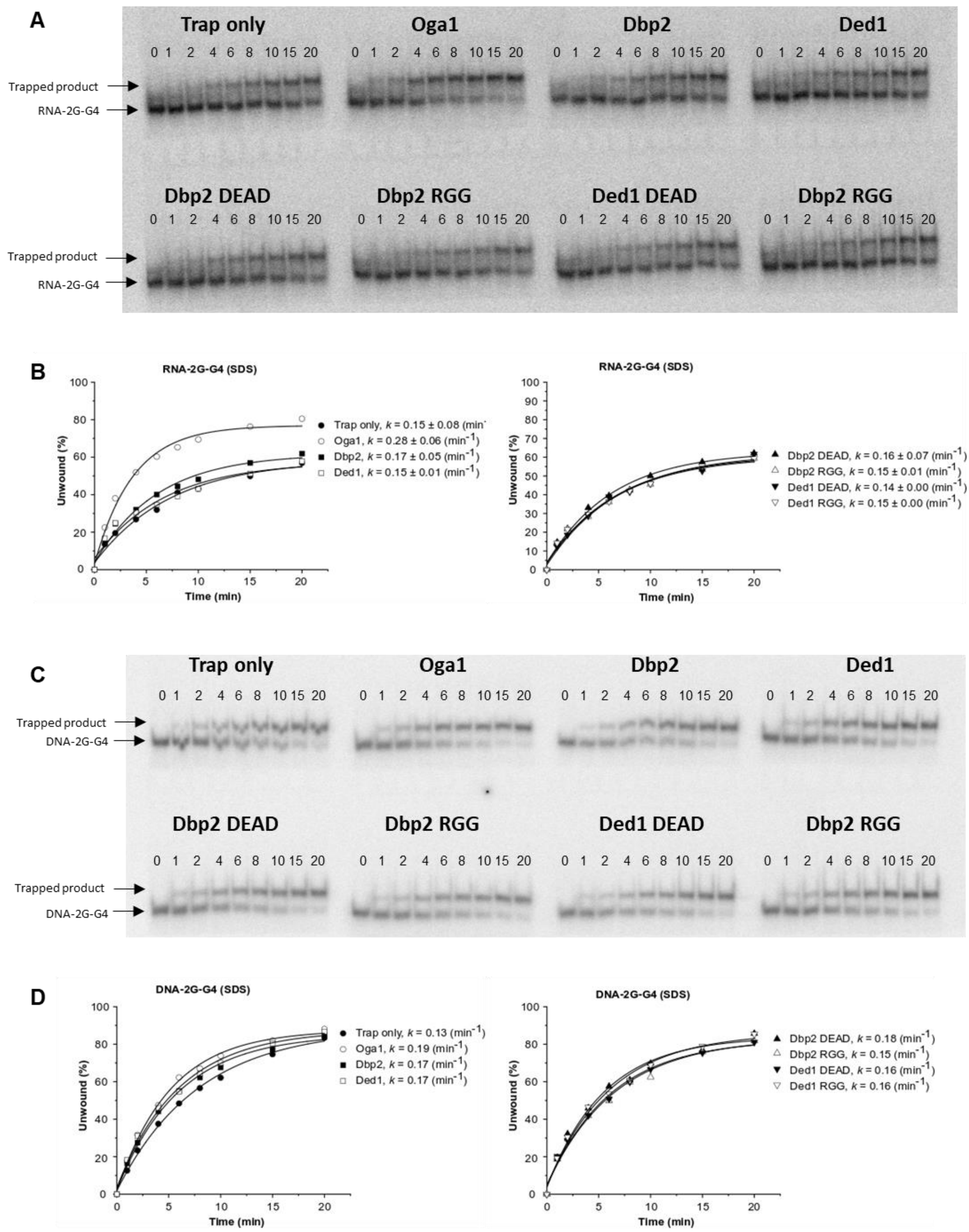

Figure S9. Boiling in SDS eliminates the G4-destabilizing activity of Dbp2 and Ded1. **(A)** Helicase trap assays with RNA-2G-G4. (**B**) The amount of unwound G4 RNA was quantified in two different experiments, and the average amount was fitted to a monoexponential function. *k* values show mean of two independent experiments ± SD. **(C)** Helicase trap assays with DNA-2G-G4 using the indicated protein variants at different time points. **(D)** The amount of unwound G4 DNA based on the gels in C) was quantified and fitted to a monoexponential function to determine *k* values. n.p. indicates not possible to determine the *k*.

SUPPORTING TABLES

Name	Sequence (5'-3')
Oga1-R249-259A-fwd	GCTCGTCCTGCTGCCGGTGGCCGTCCTAACCGTGCTCCCCGTGCTGGTCCTTCAG
Oga1-R249-259A-rev	CTGAAGGACCAGCACGGGGAGCACGGTTAGGACGGCCACCGGCAGCAGGACGAGC
Dbp2-R35-39A-fwd	GGATACCGCAATAACTATAGTGCTGGCGGCGGTGCTGGAGGTTTCA
Dbp2-R35-39A-rev	TGAAACCTCCAGCACCGCCGCCAGCACTATAGTTATTGCGGTATCC
Dbp2-E277Q-fwd	GTGTGACCTATTTAGTGCTGGATCAGGCCGACCGAATG
Dbp2-E277Q-rev	CATTCGGTCGGCCTGATCCAGCACTAAATAGGTCACAC
Dbp2-R510-516-521-526A-fwd	CAAGCGGTGGTGCTGGTGGTAACTACCGCGCTGGCGGCTATGGTGCTGGTGGTTTCCGTGCTGG AGGAGGTTATGG
Dbp2-R510-516-521-526A-rev	CCATAACCTCCTCCAGCACGGAAACCACCAGCACCATAGCCGCCAGCGCGGTAGTTACCACCAGC ACCACCGCTTG
Ded1-E337Q-fwd	AACATTAAGTTTTTGGTGTTGGATCAGGCTGATCGTATGCTGGATATG
Ded1-E337Q-rev	CATATCCAGCATACGATCAGCCTGATCCAACACCAAAAACTTAATGTT
Ded1-R574-581-584-fwd	GGTGGTAATGGAGCTGGTGGCCGTTACAGTGGTGCTGGTGGTGCTGGAGGCAATGC
Ded1-R574-581-584-rev	GCATTGCCTCCAGCACCACCAGCACCACTGTAACGGCCACCAGCTCCATTACCACC

Table S1. List of primers used for the mutagenesis.

Accession #	Gene	Description	Experiment	G4	scr	m4
**O13370**	ded1	ATP-dependent RNA helicase ded1	1	2625	275	176
			2	1372	587	408
			3	2895	374	1015
**P24782**	dbp2	ATP-dependent RNA helicase dbp2	1	1683	238	211
			2	1243	742	691
			3	2166	188	648
O42947	SPBC16H5.12c	Uncharacterized protein C16H5.12c	1	1437	0	0
			2	689	489	366
			3	708	60	56
Q10490	lrs1	Putative leucine--tRNA ligase, cytoplasmic	1	1680	0	0
			2	268	0	0
			3	415	0	0
Q9UUG1	SPAC926.08c	Ribosome production factor 2 homolog	1	469	0	0
			2	292	0	0
			3	569	0	0
P32747	ura3	Dihydroorotate dehydrogenase (quinone), mitochondrial	1	318	0	0
			2	81	0	0
			3	803	123	172
**O42914**	oga1	Stm1 homolog oga1	1	403	0	193
			2	295	121	0
			3	480	137	0
P0CT54	tef102	Elongation factor 1-alpha-B	1	1123	0	0
			2	0	0	0
			3	0	0	0
O14007	cbf5	H/ACA ribonucleoprotein complex subunit 4	1	441	0	0
			2	173	144	0
			3	473	72	0
**O13622**	mss116	ATP-dependent RNA helicase mss116, mitochondrial	1	0	0	0
			2	359	131	117
			3	645	98	188
P0CT53	tef101	Elongation factor 1-alpha-A	1	0	0	0
			2	260	133	158
			3	726	225	192
O14253	cbc1	Nuclear cap-binding protein subunit 1	1	402	0	0
			2	465	0	0
			3	0	0	0
**Q09871**	SPAC12G12.07c	Uncharacterized protein C12G12.07c	1	830	0	0
			2	0	0	0
			3	0	0	0
O14217	tom70	Probable mitochondrial import receptor subunit tom70	1	93	0	0
			2	211	0	0
			3	437	0	76
O94514	nop56	Nucleolar protein 56	1	158	0	0
			2	209	51	0
			3	325	0	0
Q09330	mlo3	mRNA export protein mlo3	1	146	0	0
			2	250	129	72
			3	286	138	102
Q09188	anc1	ADP,ATP carrier protein	1	139	0	0
			2	0	0	0
			3	463	282	281
O60164	ppp1	Pescadillo homolog	1	556	0	0
			2	0	0	0
			3	0	0	0
P56286	tif211	Eukaryotic translation initiation factor 2 subunit alpha	1	113	0	0
			2	321	107	0
			3	101	214	239
**O94236**	moe1		1	512	0	0

		Eukaryotic translation initiation factor 3 subunit D	2	0	0	0
			3	0	0	0
O94284	hmt2	Sulfide:quinone oxidoreductase, mitochondrial	1	281	0	0
			2	129	0	0
			3	100	0	0
**Q9HGL3**	sum2	Protein sum2	1	445	0	0
			2	0	0	0
			3	0	0	0
O13802	ebp2	Probable rRNA-processing protein ebp2	1	0	0	0
			2	207	70	0
			3	232	0	0
O74760	tif32	Eukaryotic translation initiation factor 3 subunit A	1	432	0	0
			2	0	0	0
			3	0	0	0
P36629	prp2	Splicing factor U2AF 59 kDa subunit	1	0	0	0
			2	101	0	0
			3	329	0	0
O74777	mis3	KRR1 small subunit processome component homolog	1	0	0	0
			2	333	84	0
			3	89	0	0
P87058	sla1	La protein homolog	1	0	0	0
			2	386	0	0
			3	0	0	0
O13920	mdm28	LETM1 domain-containing protein mdm28, mitochondrial	1	156	0	0
			2	88	0	0
			3	143	0	0
Q9P7T8	SPAC694.02	Uncharacterized helicase C694.02	1	0	0	0
			2	109	0	0
			3	274	0	0
**Q09181**	ste13	Putative ATP-dependent RNA helicase ste13	1	197	0	0
			2	148	0	0
			3	0	0	0

Table S2. List of hit proteins showing the Mascot scores of the G4 sample and the non-G4 control samples (scr, m4) from three different types of pull-down experiments (experiment 1: total protein extract with rDNA oligo; experiment 2: nuclear protein extract with rDNA oligo; experiment 3: nuclear protein extract with 10A-rDNA oligo). Proteins were ranked and listed by decreasing average Mascot score. Ribosomal proteins as well as proteins with an average score lower than 100, or with a non-G4 score higher than the G4 score, are not included in the list. Proteins with putative RGG motifs are indicated in bold. Proteins with the GO term annotation “nucleic acids binding” are underlined.

GO molecular function complete	#genes total	#genes in list	expected	Fold enrichment	raw P value	FDR
translation initiation factor activity	35	4	.20	20.26	5.74E-05	1.27E-02
RNA helicase activity	45	5	.25	19.84	6.88E-06	2.10E-03
translation factor activity, RNA binding	56	6	.31	19.22	8.91E-07	3.62E-04
translation regulator activity, nucleic acid binding	62	6	.33	17.96	1.55E-06	5.40E-04
translation regulator activity	71	7	.37	16.35	1.62E-07	7.90E-05
mRNA binding	90	6	.47	10.74	1.18E-05	3.19E-03
helicase activity	88	5	.50	9.92	1.43E-04	2.90E-02
RNA binding	517	19	2.87	6.27	1.09E-12	2.67E-09
nucleic acid binding	873	19	4.93	3.65	9.37E-09	1.14E-05
heterocyclic compound binding	1499	23	8.22	2.56	3.62E-08	2.95E-05
organic cyclic compound binding	1509	23	8.27	2.54	4.15E-08	2.53E-05

Table S3. GO molecular function complete obtained from PANTHER overrepresentation test (released 20210224; http://www.pantherdb.org). FDR P < 0.05 29 of the total 30 proteins except Q09871 were mapped.
#genes total: the number of genes in the *S. pombe* reference list that map to this particular annotation data category; #genes in list: the number of genes in of the total 29 that map to this annotation data category; expected: the expected value which is the number of genes that would be expected in the list for this category, based on the *S. pombe* reference list; fold enrichment: fold enrichment of the genes observed among the 29 genes over the expected; raw P value: the raw p-value as determined by Fisher's exact test. FDR: False Discovery Rate as calculated by the Benjamini-Hochberg procedure. Annotation version and release date GO ontology database DOI: 10.5281/zenodo. 4735677 Released 20210501.

Strains	Mating type	Genotype	Source
SAK27	h+	ade6-M210 leu1 bfr1::hygr pmd1::natr	(Kawashima *et al.*, 2012)
YKY37	h-	leu1-32 ded1-1D5 bfr1::hygr pmd1::natr	This study
YKY38	h+	leu1-32 ded1-1D5 bfr1::hygr pmd1::natr	This study
YKY39	h-	leu1-32 ade6-M216 oga1::kanR bfr1::hygr pmd1::natr	This study
YKY40	h-	leu1-32 ade6-M216 oga1::kanR bfr1::hygr pmd1::natr	This study

Table S4. List of *S. pombe* strains used in this study.

References

S. A. Kawashima, A. Takemoto, P. Nurse, and T. M. Kapoor, "Analyzing fission yeast multidrug resistance mechanisms to develop a genetically tractable model system for chemical biology," *Chem. Biol.*, vol. 19, no. 7, pp. 893–901, Jul. 2012, doi: 10.1016/j.chembiol.2012.06.008.
